# Tumor mutation burden in connection with immune-related survival in uterine corpus endometrial carcinoma

**DOI:** 10.1186/s12935-021-01774-6

**Published:** 2021-01-28

**Authors:** Ling Zhao, Xueshu Fu, Xiling Han, Yanjun Yu, Yaping Ye, Jun Gao

**Affiliations:** 1 Department of Gynecology, Second Affiliated Hospital of Guizhou Medical University, Qiandongnan Second People’s Hospital, Kaili, 556000 Guizhou China; 2 Department of Gynecology, Huaian Maternity and Child Health Care Hospital, Huaian, 223002 Jiangsu China; 3 Department of Obstetrics and Gynecology, Clinical Medical College of Yangzhou University, Yangzhou, 225001 Jiangsu China; 4 Department of Gynecologic Oncology, Dalian Medical University, Dalian, 116000 Liaoning China; 5 Department of Obstetrics and Gynecology, Northern Jiangsu People’s Hospital, 98 W Nantong Rd, Yangzhou, 225001 Jiangsu China

**Keywords:** Tumor mutation burden, Endometrial carcinoma, Immune-related survival, TCGA

## Abstract

**Background:**

UCEC is the most common gynecological malignancy in many countries, and its mechanism of occurrence and development is related to tumor mutation burden (TMB) and immune cell infiltration. Therefore, it is necessary to systematically explore the TMB-related gene profile in immune cells to improve the prognosis of UCEC.

**Methods:**

We integrated TMB-related genes with basic clinical information of UCEC patients based on TCGA dataset. Differentially expressed genes (DEGs) were selected through differential expression screening, PPI, and enrichment analysis. Additionally, we analyzed the components of immune cell infiltration of the DEGs to obtain the differential immunity-related genes. A single factor and multifactor Cox regression analyses were conducted to establish new prognostic indicators of OS and DFS based on TMB-related immune genes. To further study the correlation between survival and immune cell infiltration, a Cox model based on these immune infiltration compositions was built. Using the clinical variables, we established nomograms for OS and DFS.

**Results:**

393 DEGs were significantly associated with clinical outcomes and the immune component in patients with UCEC. Gene Ontology (GO) and Kyoto Encyclopedia of Genes, Genomes (KEGG) pathway and protein-protein interaction network (PPI) analyses revealed the role of these genes and information on related pathways. Then, two prognostic models were established based on the differential immune genes for OS (GFAP and MX2) and DFS (MX2, GFAP, IGHM, FGF20, and TRAV21). In DFS, the differential immune genes were related to CD4+ T cell, CD8+ T cell, macrophage, and neutrophil (all P < 0.05). B cell and CD8+ T cell were independent prognostic factors from among the immune cell elements in UCEC. Finally, the risk scores of these models were combined with the clinical elements-based nomogram models, and the AUC values were all over 0.7.

**Conclusions:**

Our results identified several clinically significant differential immune genes and established relevant prognostic models, providing a basis for the molecular analysis of TMB and immune cells in UCEC, and identified potential prognostic and immune-related genes for UCEC. We added clinical related conditions for further analysis to confirm the identity of the genes and clinical elements-based models.

## Introduction

Uterine corpus endometrial carcinoma (UCEC) is a common type of gynecological tumor. It is estimated that there will be 65,620 new cases and 12,590 deaths caused by the disease in the United States in 2020, second only to ovarian cancer. At the same time, the mortality rate of UCEC has risen over the past 10 years [1]. The 5-year survival rate of stage I UCEC exceeds 90%, while stage IV can only reach 20% [2]. Traditional treatment methods mainly include surgery, radiotherapy, chemotherapy, and hormone therapy. For patients with metastases, surgery, and radiotherapy cannot achieve a satisfactory treatment level. These patients are treated using chemotherapy or hormone therapy, which also causes more significant damage to normal human cells and results in recurrence. Most importantly, options are often limited after traditional first-line treatment for advanced patients with metastases or recurrence, which has prompted many scholars to develop new treatment methods.

In recent years, immunotherapy has become an effective method for treating advanced cancer [3]. For example, typical clinical trials include lung cancer [4, 5] and melanoma [5–7]. Among these immunotherapies, the most important one is Immune Checkpoint Inhibitor (ICI), and its main inhibitory points include Cytotoxic T-lymphocyte antigen 4 (CTLA-4) and Programed cell death 1 receptor (PD-1) [7, 8]. More specifically, ICI blocks the checkpoint activity of T cells, which increases the sensitivity of immune cells to the recognition of cancer cells and triggers the immune system to attack and destroy cancer cells. Tumor mutation burden (TMB) refers to the total number of mutations per mega base in tumor tissue. There is increasing evidence that the burden of tumor mutations is related to immunotherapy in cancer patients [9]. Although most inhibitors are rarely clinically approved for humans, their immunotherapy has shown great potential in refractory tumors. Immunotherapy research has also been conducted on targeted therapy for UCEC. The level of TMB and microsatellite instability can predict whether patients with UCEC may benefit from immunotherapy [10, 11]. Uterine carcinosarcoma is a rare and aggressive histological variant of UCEC with a poor prognosis [10]. Bhangoo et al. [11] found that inactivation of DNA polymerase ɛ (POLE) mutation is associated with a high TMB and ICI in carcinosarcoma. In summary, the study of TMB on immune invasiveness of UCEC requires further research. Therefore, we aimed to study the relationship between the clinical prognosis of UCEC and the role of TMB and immunity.

## Materials and methods

### Data collection

Clinical data on a UCEC cohort, including age, stage, grade, histological type, together with transcriptome data, were obtained from the TCGA data portal (https://portal.gdc.cancer.gov/). Meanwhile, we downloaded the processed “mask somatic mutation” data using the VarScan algorithm in the TCGA database. The R package “maftools function” [12] was adopted to describe mutated genes in the UCEC samples. Since each dataset was retrieved from published reports, it was verified that written informed consent had been obtained.

### TMB score and clinical characteristics

We used Perl script (JAVA8 platform) to remove silent mutations from the samples while calculating the number of base mutations and then corrected it to the number of base mutations per million bases, which was the TMB value. The samples were divided into a low group and high group based on the median value of TMB. Further, we used Kaplan-Meier statistics to analyze survival differences between these two groups. The Wilcoxon rank-sum test was used to evaluate the correlation between TMB and various clinical variables. Furthermore, X-tile software (Yale University, New Haven, Connecticut, USA) was utilized to select the optimal threshold value for age. We analyzed overall survival (OS) and disease-free survival (DFS) to determine the prognostic value of the two TMB groups of patients with UCEC.

### Differentially expressed genes and enrichment analysis

The Limma package of R software was adopted to predict differentially expressed genes (DEGs) between the high-TMB group and low-TMB group. Genes that exhibited a |Fold Change|of > 1 with an adjusted P-value of < 0.05 were regarded as significant DEGs. Then, the “pheatmap” package of R software was used to assess the effect of the difference by constructing a heatmap plot. Kyoto Encyclopedia of Genes and Genomes (KEGG) and Gene Ontology (GO) enrichment analyses were carried out to identify the primary biological attributes for the genes selected above using the “enrichplot,” “clusterProfiler,” “ggplot2” and “org.Hs.eg.db” functions of R software. Biological processes, cellular components, and molecular functions are the three primary functional components of GO enrichment analysis. KEGG analysis can identify the neighbor pathways of the DEGs.

### Search tool for the retrieval of interacting genes database (STRING)

STRING (https://string-db.org/) is a database of known and predicted protein-protein interactions, and its latest version, 11.0, includes 24,584,628 proteins from 5090 organisms [13]. This tool was applied to explore the physical and functional associations between the DEGs and conduct the protein-protein interaction network (PPI) analysis of the DEGs. The results of the PPI analysis provided the number of adjacent nodes per gene, which was obtained through a histogram created using R software.

### Gain differential immune genes and survival analysis

CIBERSOR is a general calculation method used to quantify cellular components from many tissue gene expression profiles (GEP), which could accurately estimate the immune composition of tumor biopsy [14]. We applied the immune cell expression profiles to the “CIBERSORT” algorithm to determine immune cell content in the UECE sample. The vioplot package was used to determine the expression differences of various immune cells in the high-TMB and low-TMB groups. After intersecting the immune cell-related genes derived from the immune database (https://www.immport.org/) and the DEGs, the differential immune genes were obtained using the “VennDiagram” package. Kaplan–Meier survival analysis was performed to assess the survival of the immune-related genes.

### Establishment of differential immune genes-based prognostic index models

First, differential immune genes were extracted to be further study using a Cox single-factor and multifactor regression analysis for OS and DFS, which was performed using R software. The statistical indicators that were used were: hazard ratio (HR), 95% confidence interval (CIs), and log-rank P-value (a P-value of < 0.05 was considered statistically significant). The receiver operating characteristic (ROC) curve of each model was constructed. Furthermore, patients were divided into high-risk and low-risk groups based on the median risk score.

### TIMER database

TIMER web server (https://cistrome.shinyapps.io/timer/) is a comprehensive resource for the systematical analysis of six immune infiltrates, including B cells, CD4+ T cells, CD8+ T cells, Neutrophils, Macrophages, and Dendritic cells, across diverse cancer types [15]. Moreover, the survival module in the TIMER database was also used to draw Kaplan–Meier plots for the immune infiltrates and hub immune genes to visualize survival differences. The P-value of the log-rank test used to compare the survival curves of the two groups is shown in each plot.

### Outputs of the immune elements-based models

Surv (UCEC) ~ B_cell + CD8_Tcell + CD4_Tcell + Macrophage + Neutrophil + Dendritic was the formula of Cox’s regression model. This model was fitted using the “coxph” function of the R software package, “survival.” HR is presented as lower and upper 95% confidential intervals, and the threshold p-value was set at 0.05.

### Construction of nomogram models for OS and DFS

The information on clinical characteristics obtained from public datasets and two risk groups were collected and divided into a modeling group and a verification group based on a 7:3 ratio. The “rms” package of R software was used to construct the nomogram and calibration plots. ROC curves were also plotted to determine the sensitivity and specificity of the nomogram models.

### Statistical analysis

R 3.6.3 (https://www.r-project.org/) software was used for all statistical analyses. Additionally, the R package “survival” was utilized to conduct the univariate Cox regression analysis to examine the relationship between OS and DFS with gene expression. At the same time, a model was constructed based on the multivariate Cox regression analysis. X-tile software was used to group patient data based on age, with 69 years of age for OS and 59 years of age for DFS used the critical values (Figs. 3, 4). Then, nomogram plots were conducted for both clinical parameters and risk groups. The area under the curve (AUC) of the ROC curve was computed using the ROC function of the “survival” package in R software. A P value of < 0.05 was considered to indicate statistical significance.

## Results

### Analyze mutation data in UCEC

**Fig. 1:**
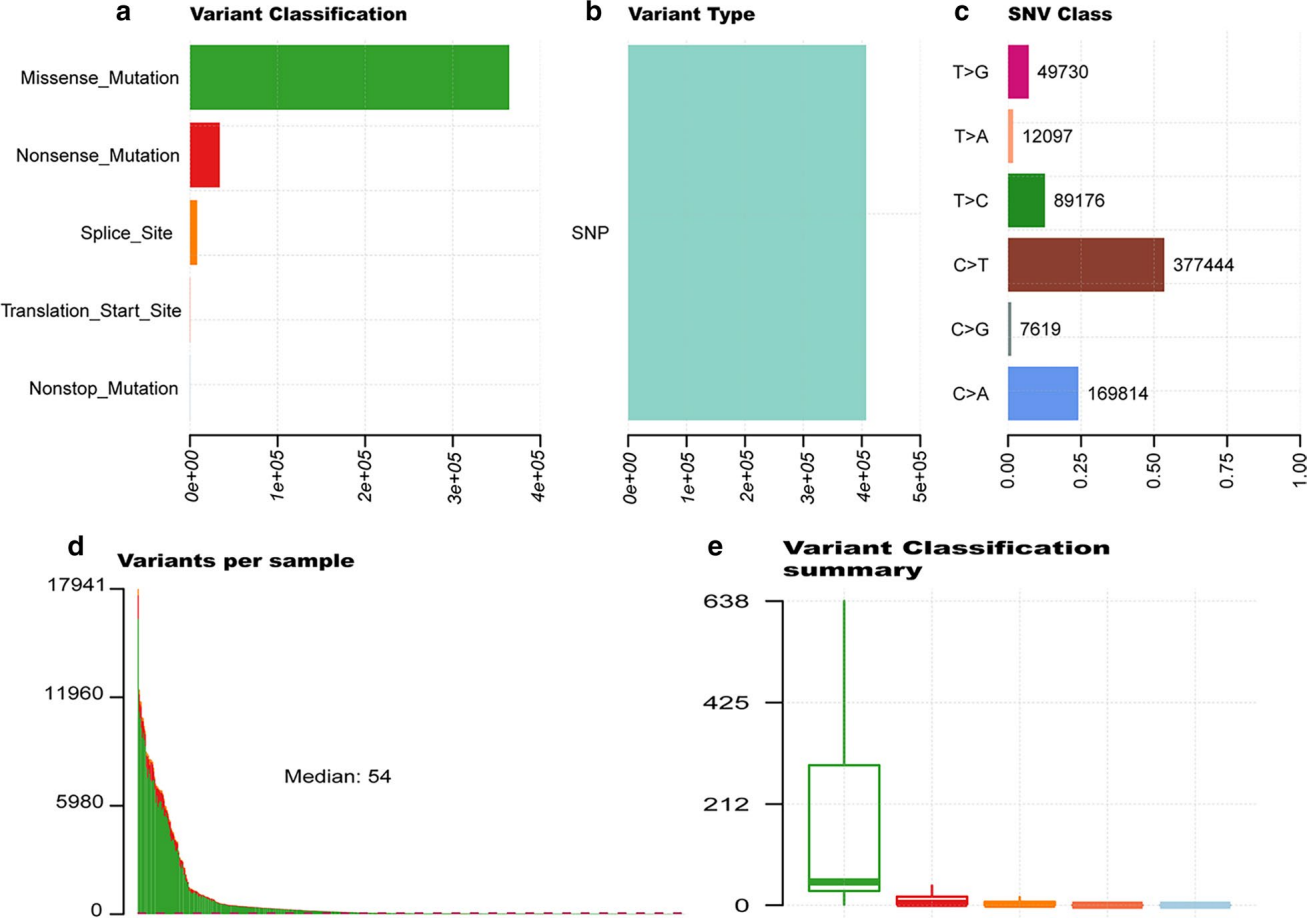
Summary description of gene mutations in UCEC. **a** Statistics of the number of variant classifications: missense mutation, nonsense mutation, splice site, translation start site, and nonstop mutation; **b** counting of variant types: only SNPs in UCEC; **c** summary of base mutations: T>G, T>A, T>C, C>T, C>G and C>A. **d**, **e** TMB in tissues; **f** top 10 mutated genes

**Fig. 2:**
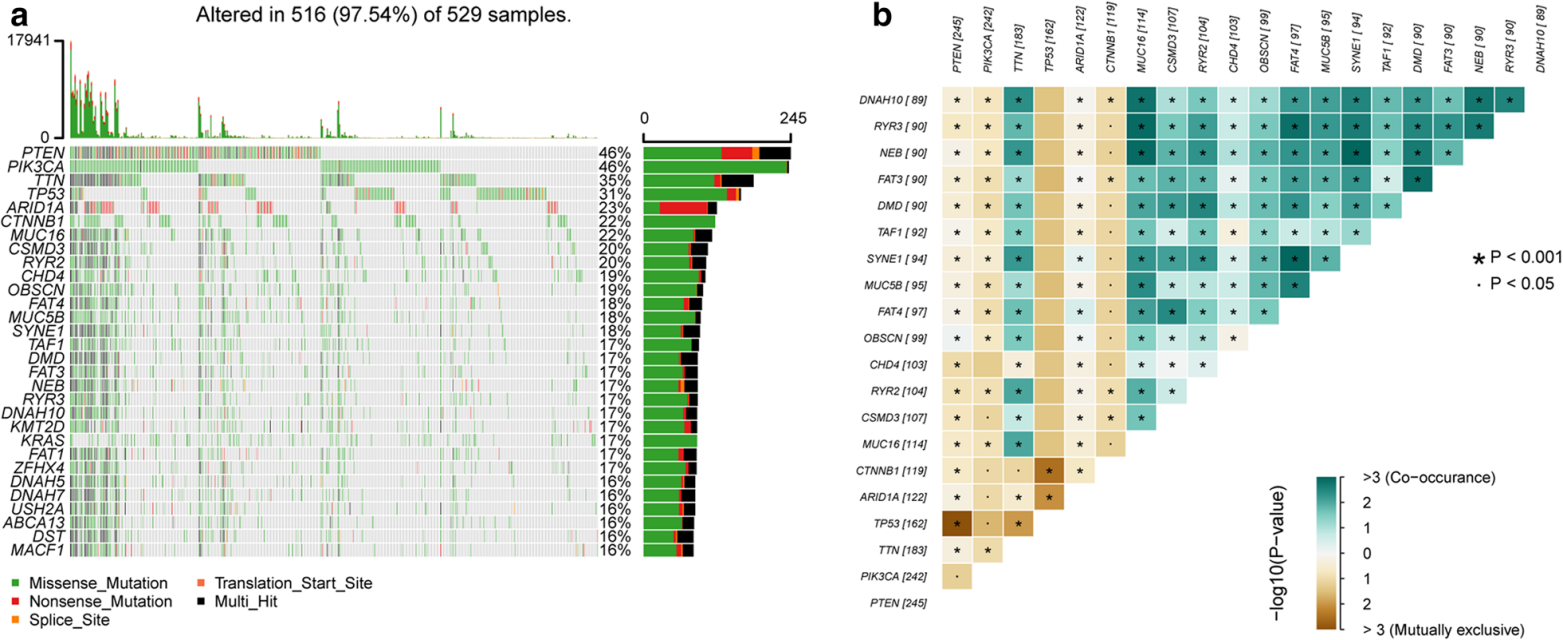
Display of mutation information in UCEC. **a** The waterfall of mutation information: the ordinate on the left indicates the name of the gene, and the abscissa represents different samples. Six different colors were used to show the six different types of mutations: gray color indicated no mutation, and the icon shows the color that represents the remaining five types of mutations; **b** relationship between pairwise mutant genes: green color indicates a positive correlation and a brown color represents a negative correlation

The somatic mutation data of 375 UCEC patients were downloaded from the TCGA database, and these materials were visualized using the “maftools” R package. Diagrams a–c in Fig. 1 provides a summary of the types of mutations in the samples, in which missense mutations occupied the leading position (Fig. 1a), C>T was the most common single nucleotide variant (SNV) (Fig. 1c). Only one mutation type, Single Nucleotide Polymorphism (SNP), was found in UCEC (Fig. 1b). The mutation value and median of each sample are shown in Fig. 1d, e. Furthermore, we showed the top 10 mutated genes in the samples, which included TTN (35%), MUC16 (22%), PTEN (46%), RYR2 (20%), CSMD3 (20%), PIK3CA (46%), TP53 (31%), ARID1A (23%), CHD4 (19%) and CTNNB1 (22%) (Fig. 1f). The waterfall chart shown in Fig. 2a demonstrates the specific mutation types and proportions of the mutant genes in each sample. Figure 2b shows the connection between two mutant genes, in which the green color indicates a positive correlation, while a brown color indicates a negative correlation.

### Relationship between TMB and clinical features

**Fig. 3:**
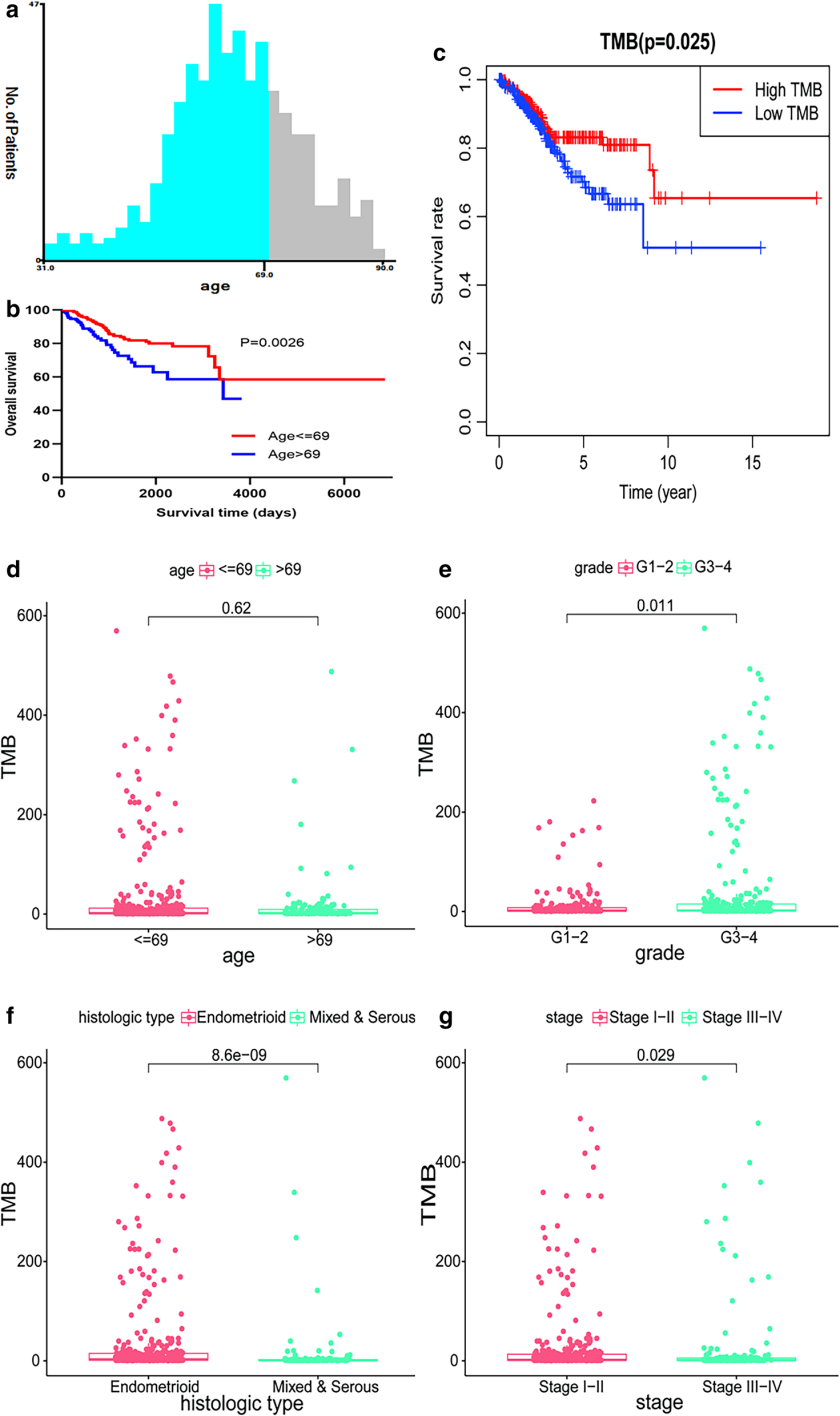
Connection between TMB and clinical variables for OS. **a**, **b** Age grouping using X-tile in OS; **c** survival analysis of the high-TMB and low-TMB groups; **d**–**g** the link between TMB and clinical features, including age, grade, histological type, and stage

**Fig. 4:**
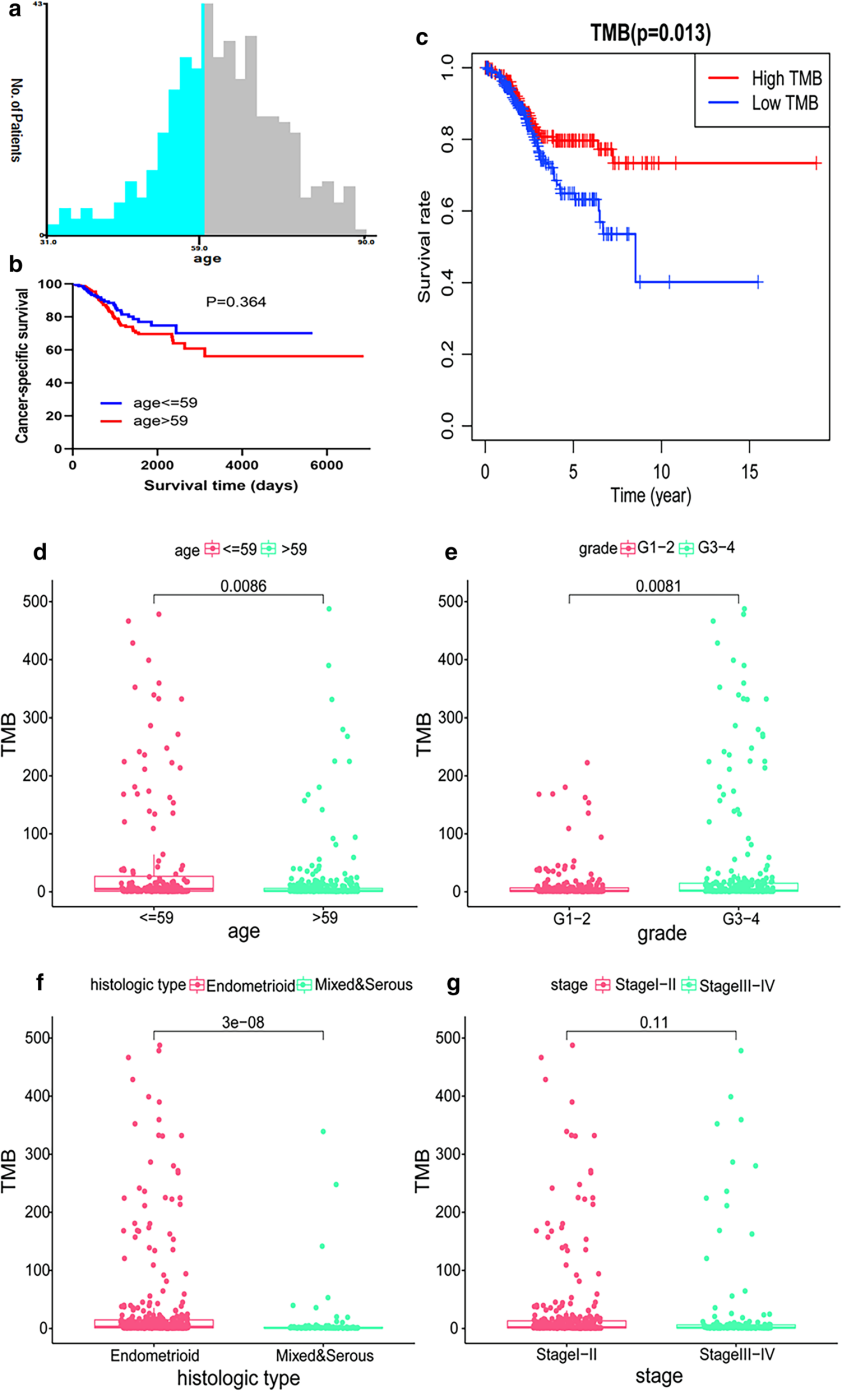
Relationship between TMB and clinical characteristics for DFS. **a**, **b** Age grouping using X-tile in DFS; **c** survival analysis of the high-TMB and low-TMB groups; **d**–**g** the link between TMB and clinical features, including age, grade, histological type, and stage

The TMB of UCEC cases was calculated to determine its clinical significance. For OS, initially, we found that 69 years of age was the best age cut-off value using x-tile software, and the median value of TMB was used as the optimal cut-off value to divide these samples into two groups: low and high TMB groups. It could be detected that the low TMB group showed a more unsatisfactory survival outcome compared to the high TMB group, based on values in the log-rank test and Kaplan–Meier curve (P = 0.025, Fig. 3c). The high TMB group was closely related to the grade 3 or 4 (P = 0.011, Fig. 3e) endometroid pathological type (P < 0.001, Fig. 3f) and stage I or II (P = 0.029, Fig. 3g), while there was no significant difference between TMB and age (P = 0.62, Fig. 3d). For DFS, Fig. 4 shows that 59 years of age was the optimal threshold and that the association between TMB and survival were similar to the results for OS. Additionally, expression levels of TMB decreased due when age was ≤ 59, the grade was 3–4, and endometrioid histological type (all P < 0.05), and there were no significant associations between TMB and stage (P = 0.11).

### Enrichment analyses and the PPI of genes in the different TMB group

**Fig. 5:**
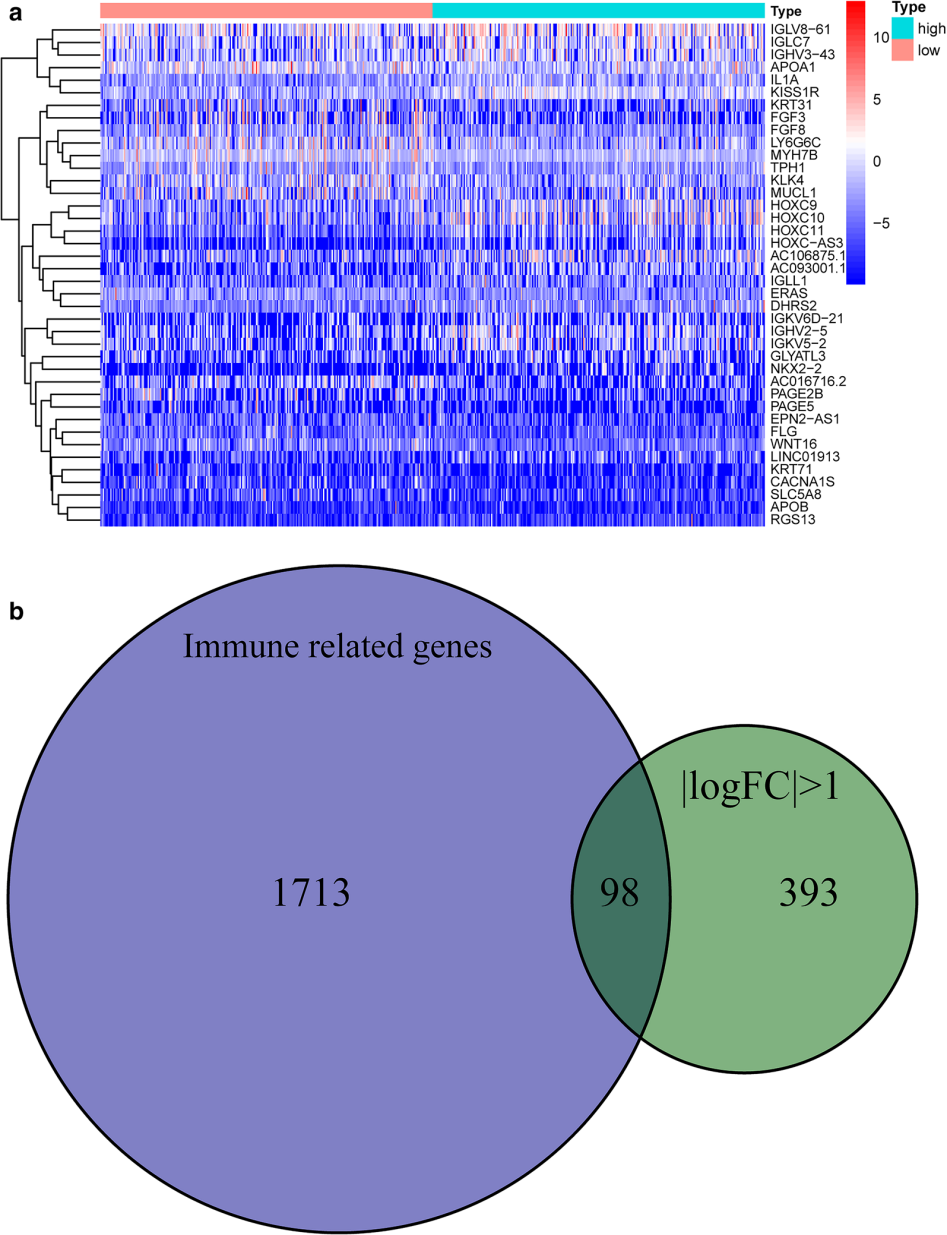
Heatmap and enrichment analysis of the DEGs. **a** Distribution of genes in the high and low TMB groups: the red color represents a higher level of expression, and blue color represents a lower level of expression; **b** Venn diagram of the immune-related genes and DEGs

**Fig. 6:**
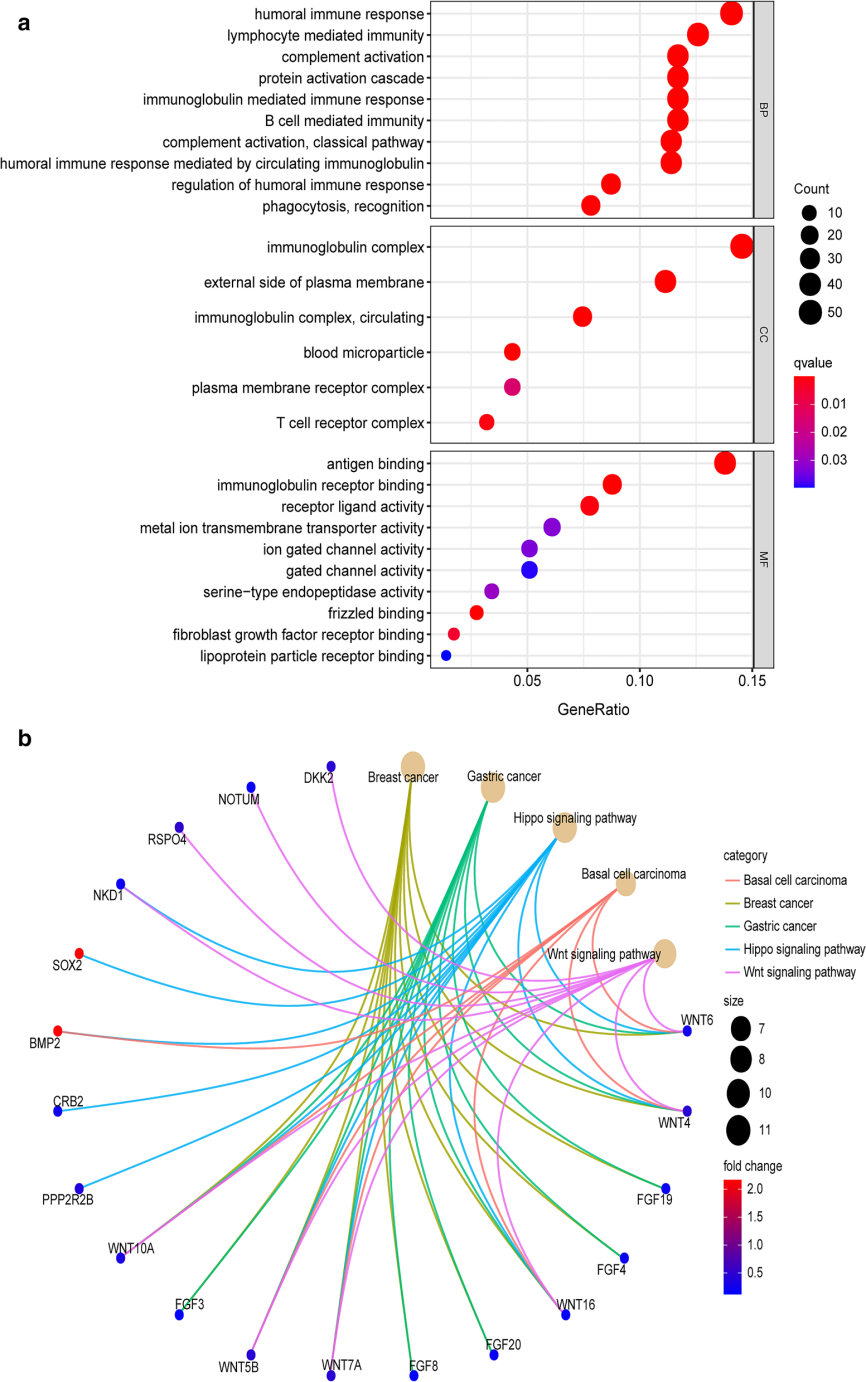
Enrichment analysis of the DEGs. **a** GO plots of the DEGs. **b** KEGG analysis results of the DEGss

We found 393 differentially expressed genes (DEGs) between the high TMB and low TMB groups, and the top 19 DEGs are shown in Table 1. The heatmap constructed shows the expression levels of the DEGs (Fig. 5a). The Venn diagram was created to show 1713 immune-related genes and 393 DEGs to obtain 98 differential immune genes (Fig. 5b). After that, the KEGG pathway and GO term enrichment analyses of these DEGs were conducted. Figure 6a and Table 2 show that the GO terms primarily enriched for biological processes included humoral immune response and lymphocyte-mediated immunity. Simultaneously, the Immunoglobulin complex, external side of the plasma membrane, and immunoglobulin complex were the cellular components that were mainly enriched.

**Table 1:** The top 19 DEGs in UCEC

Gene	Low group	High group	logFC	P-value	FDR
ERVMER34-1	8.007284856	2.261724965	− 1.823889628	0.000229986	0.001203484
CRYGN	0.421748475	0.208045874	− 1.019481175	7.82E−11	4.35E−09
IGKV2OR22-4	0.446894633	1.119245354	1.324519704	0.010006045	0.026333049
MYT1	0.28577249	0.12531108	− 1.189353053	3.48E−08	7.38E−07
IGHV4-31	8.410528443	17.48325048	1.05570508	6.91E−05	0.00044633
SIX3	2.3926374	0.838171842	− 1.513283815	0.001384316	0.005254444
PAGE2	4.185109335	2.030715895	− 1.043276898	0.000968098	0.003926813
AC011008.2	0.31787423	0.150634816	− 1.0774008	1.59E−07	2.63E−06
CYP4F3	0.403131219	0.823924444	1.031262531	0.00367986	0.011629492
COL22A1	1.05899893	0.495353661	− 1.096170312	0.00192648	0.006859294
GRIK5	2.776782427	1.332647212	− 1.05911923	7.20E−05	0.000460392
TTYH1	0.84036637	0.407639698	− 1.043723874	0.002854537	0.009465163
TCL1A	0.149056807	0.377160857	1.339317699	0.00311425	0.010161885
IGLV5-45	13.93991507	28.93674286	1.053680768	0.000152768	0.000861658
HMGN2P6	0.199381466	0.404795154	1.021660709	0.01678836	0.03972772
CYTL1	5.128458024	1.579728719	− 1.698848284	1.13E−12	1.26E−10
HTR1E	0.400763548	0.090015782	− 2.154501428	0.000105609	0.000635234
IGLL1	0.189352489	0.900723467	2.250009862	0.0085663	0.023218884
HOXC8	0.283649843	0.703358842	1.310149851	5.47E−05	0.000365756

**Table 2:** Detailed GO profiles of the DEGs in UCEC

	ID	Description	GeneRatio	BgRatio	p.adjust	qvalue	geneID	Count
BP	GO:0006958	Complement activation, classical pathway	38/337	137/18670	4.55E−31	4.27E−31	IGHV4-31/IGLL1/IGHV4-34/IGLV1-44/IGHM/IGKV2-30/IGKV5-2/IGHG4/IGLV2-8/IGHV3-43/IGLV6-57/IGHV3-53/IGHV3-72/IGHV3-66/IGLV3-21/IGHV4-59/IGHV3-20/IGHV3-23/IGHV3-73/IGLV2-14/IGHV3-13/IGKV1-5/IGHV2-26/IGHG3/IGHV2-70/IGHV1-69/IGLV3-19/IGLL5/IGLV7-43/IGHV3-30/IGHV3-64/IGKV1-17/IGKV1D-12/IGKC/IGLC7/IGHV3-74/IGHG2/IGHV2-5	38
BP	GO:0002455	Humoral immune response mediated by circulating immunoglobulin	38/337	150/18670	9.97E−30	9.37E−30	IGHV4-31/IGLL1/IGHV4-34/IGLV1-44/IGHM/IGKV2-30/IGKV5-2/IGHG4/IGLV2-8/IGHV3-43/IGLV6-57/IGHV3-53/IGHV3-72/IGHV3-66/IGLV3-21/IGHV4-59/IGHV3-20/IGHV3-23/IGHV3-73/IGLV2-14/IGHV3-13/IGKV1-5/IGHV2-26/IGHG3/IGHV2-70/IGHV1-69/IGLV3-19/IGLL5/IGLV7-43/IGHV3-30/IGHV3-64/IGKV1-17/IGKV1D-12/IGKC/IGLC7/IGHV3-74/IGHG2/IGHV2-5	38
BP	GO:0006956	Complement activation	39/337	175/18670	2.00E−28	1.88E−28	IGHV4-31/IGLL1/IGHV4-34/IGLV1-44/IGHM/IGKV2-30/IGKV5-2/IGHG4/IGLV2-8/IGHV3-43/IGLV6-57/IGHV3-53/IGHV3-72/CFD/IGHV3-66/IGLV3-21/IGHV4-59/IGHV3-20/IGHV3-23/IGHV3-73/IGLV2-14/IGHV3-13/IGKV1-5/IGHV2-26/IGHG3/IGHV2-70/IGHV1-69/IGLV3-19/IGLL5/IGLV7-43/IGHV3-30/IGHV3-64/IGKV1-17/IGKV1D-12/IGKC/IGLC7/IGHV3-74/IGHG2/IGHV2-5	39
BP	GO:0072376	Protein activation cascade	39/337	198/18670	2.28E−26	2.14E−26	IGHV4-31/IGLL1/IGHV4-34/IGLV1-44/IGHM/IGKV2-30/IGKV5-2/IGHG4/IGLV2-8/IGHV3-43/IGLV6-57/IGHV3-53/IGHV3-72/CFD/IGHV3-66/IGLV3-21/IGHV4-59/IGHV3-20/IGHV3-23/IGHV3-73/IGLV2-14/IGHV3-13/IGKV1-5/IGHV2-26/IGHG3/IGHV2-70/IGHV1-69/IGLV3-19/IGLL5/IGLV7-43/IGHV3-30/IGHV3-64/IGKV1-17/IGKV1D-12/IGKC/IGLC7/IGHV3-74/IGHG2/IGHV2-5	39
BP	GO:0016064	Immunoglobulin mediated immune response	39/337	218/18670	8.39E−25	7.88E−25	IGHV4-31/IGLL1/IL13RA2/IGHV4-34/IGLV1-44/IGHM/IGKV2-30/IGKV5-2/IGHG4/IGLV2-8/IGHV3-43/IGLV6-57/IGHV3-53/IGHV3-72/IGHV3-66/IGLV3-21/IGHV4-59/IGHV3-20/IGHV3-23/IGHV3-73/IGLV2-14/IGHV3-13/IGKV1-5/IGHV2-26/IGHG3/IGHV2-70/IGHV1-69/IGLV3-19/IGLL5/IGLV7-43/IGHV3-30/IGHV3-64/IGKV1-17/IGKV1D-12/IGKC/IGLC7/IGHV3-74/IGHG2/IGHV2-5	39
BP	GO:0019724	B cell mediated immunity	39/337	221/18670	1.20E−24	1.12E−24	IGHV4-31/IGLL1/IL13RA2/IGHV4-34/IGLV1-44/IGHM/IGKV2-30/IGKV5-2/IGHG4/IGLV2-8/IGHV3-43/IGLV6-57/IGHV3-53/IGHV3-72/IGHV3-66/IGLV3-21/IGHV4-59/IGHV3-20/IGHV3-23/IGHV3-73/IGLV2-14/IGHV3-13/IGKV1-5/IGHV2-26/IGHG3/IGHV2-70/IGHV1-69/IGLV3-19/IGLL5/IGLV7-43/IGHV3-30/IGHV3-64/IGKV1-17/IGKV1D-12/IGKC/IGLC7/IGHV3-74/IGHG2/IGHV2-5	39
BP	GO:0006959	Humoral immune response	47/337	356/18670	2.12E−24	1.99E−24	IGHV4-31/IGLL1/IGHV4-34/IGLV1-44/IGHM/IGKV2-30/IGKV5-2/IGHG4/IGLV2-8/IGHV3-43/IGLV6-57/IGHV3-53/REG1A/IGHV3-72/CFD/IGHV3-66/IGLV3-21/IGHV4-59/IGHV3-20/IGHV3-23/KLK7/IGHV3-73/IGLV2-14/IGHV3-13/KLK3/IFNG/IGKV1-5/IGHV2-26/IGHG3/IGHV2-70/IGHV1-69/IGLV3-19/CAMP/IGLL5/IGLV7-43/IGHV3-30/KLK5/IGHV3-64/IGKV1-17/IGKV1D-12/IGKC/CXCL9/IGLC7/IGHV3-74/CXCL13/IGHG2/IGHV2-5	47
BP	GO:0006910	Phagocytosis, recognition	26/337	84/18670	9.61E−23	9.03E−23	IGHV4-31/IGLL1/IGHV4-34/IGHM/IGHG4/IGHV3-43/IGHV3-53/IGHV3-72/IGHV3-66/IGHV4-59/IGHV3-20/IGHV3-23/IGHV3-73/IGHV3-13/IGHV2-26/IGHG3/IGHV2-70/IGHV1-69/IGLL5/IGHV3-30/IGHV3-64/IGKC/IGLC7/IGHV3-74/IGHG2/IGHV2-5	26
BP	GO:0002920	Regulation of humoral immune response	29/337	134/18670	1.10E−20	1.04E−20	IGHV4-34/IGLV1-44/IGKV2-30/IGKV5-2/IGHG4/IGLV2-8/IGLV6-57/IGHV3-53/IGLV3-21/IGHV4-59/IGHV3-23/KLK7/IGLV2-14/IGHV3-13/IGKV1-5/IGHG3/IGHV2-70/IGHV1-69/IGLV3-19/IGLV7-43/IGHV3-30/KLK5/IGKV1-17/IGKV1D-12/IGKC/IGLC7/CXCL13/IGHG2/IGHV2-5	29
BP	GO:0002449	Lymphocyte mediated immunity	42/337	352/18670	5.32E−20	5.00E−20	IGHV4-31/IGLL1/IL13RA2/IGHV4-34/IGLV1-44/IGHM/IGKV2-30/IGKV5-2/ULBP2/LAG3/IGHG4/IGLV2-8/IGHV3-43/IGLV6-57/IGHV3-53/IGHV3-72/CD8A/IGHV3-66/IGLV3-21/IGHV4-59/IGHV3-20/IGHV3-23/IGHV3-73/IGLV2-14/IGHV3-13/IGKV1-5/IGHV2-26/IGHG3/IGHV2-70/IGHV1-69/IGLV3-19/IGLL5/IGLV7-43/IGHV3-30/IGHV3-64/IGKV1-17/IGKV1D-12/IGKC/IGLC7/IGHV3-74/IGHG2/IGHV2-5	42
BP	GO:0002460	Adaptive immune response based on somatic recombination of immune receptors built from immunoglobulin superfamily domains	42/337	361/18670	1.29E−19	1.22E−19	IGHV4-31/IGLL1/IL13RA2/IGHV4-34/IGLV1-44/IGHM/IGKV2-30/IGKV5-2/ULBP2/IGHG4/IGLV2-8/IGHV3-43/IGLV6-57/IGHV3-53/IGHV3-72/CD8A/IGHV3-66/IGLV3-21/IGHV4-59/IGHV3-20/IGHV3-23/IGHV3-73/IGLV2-14/IGHV3-13/IGKV1-5/IGHV2-26/IGHG3/IGHV2-70/IGHV1-69/IGLV3-19/IGLL5/IGLV7-43/IGHV3-30/IGHV3-64/IGKV1-17/IGKV1D-12/IGKC/IGLC7/IGHV3-74/CXCL13/IGHG2/IGHV2-5	42
BP	GO:0030449	Regulation of complement activation	26/337	115/18670	4.87E−19	4.57E−19	IGHV4-34/IGLV1-44/IGKV2-30/IGKV5-2/IGHG4/IGLV2-8/IGLV6-57/IGHV3-53/IGLV3-21/IGHV4-59/IGHV3-23/IGLV2-14/IGHV3-13/IGKV1-5/IGHG3/IGHV2-70/IGHV1-69/IGLV3-19/IGLV7-43/IGHV3-30/IGKV1-17/IGKV1D-12/IGKC/IGLC7/IGHG2/IGHV2-5	26
BP	GO:2000257	Regulation of protein activation cascade	26/337	116/18670	5.70E−19	5.35E−19	IGHV4-34/IGLV1-44/IGKV2-30/IGKV5-2/IGHG4/IGLV2-8/IGLV6-57/IGHV3-53/IGLV3-21/IGHV4-59/IGHV3-23/IGLV2-14/IGHV3-13/IGKV1-5/IGHG3/IGHV2-70/IGHV1-69/IGLV3-19/IGLV7-43/IGHV3-30/IGKV1-17/IGKV1D-12/IGKC/IGLC7/IGHG2/IGHV2-5	26
BP	GO:0050853	B cell receptor signaling pathway	27/337	129/18670	6.11E−19	5.74E−19	IGHV4-31/IGLL1/IGHV4-34/IGHM/IGHG4/IGHV3-43/IGHV3-53/IGHV3-72/IGHV3-66/IGHV4-59/IGHV3-20/IGHV3-23/IGHV3-73/IGHV3-13/IGHV2-26/IGHG3/IGHV2-70/IGHV1-69/IGLL5/IGHV3-30/IGHV3-64/IGKC/IGLC7/CD22/IGHV3-74/IGHG2/IGHV2-5	27
BP	GO:0006911	Phagocytosis, engulfment	26/337	118/18670	7.88E−19	7.41E−19	IGHV4-31/IGLL1/IGHV4-34/IGHM/IGHG4/IGHV3-43/IGHV3-53/IGHV3-72/IGHV3-66/IGHV4-59/IGHV3-20/IGHV3-23/IGHV3-73/IGHV3-13/IGHV2-26/IGHG3/IGHV2-70/IGHV1-69/IGLL5/IGHV3-30/IGHV3-64/IGKC/IGLC7/IGHV3-74/IGHG2/IGHV2-5	26
BP	GO:0006909	Phagocytosis	41/337	369/18670	1.63E−18	1.53E−18	IGHV4-31/IGLL1/IGHV4-34/IGLV1-44/IGHM/IGKV2-30/IGKV5-2/SIRPG/IGHG4/IGLV2-8/IGHV3-43/APOA1/IGLV6-57/IGHV3-53/IGHV3-72/IGHV3-66/IGLV3-21/IGHV4-59/IGHV3-20/IGHV3-23/IGHV3-73/IGLV2-14/IGHV3-13/IFNG/IGKV1-5/IGHV2-26/IGHG3/IGHV2-70/IGHV1-69/IGLV3-19/IGLL5/IGLV7-43/IGHV3-30/IGHV3-64/IGKV1-17/IGKV1D-12/IGKC/IGLC7/IGHV3-74/IGHG2/IGHV2-5	41
BP	GO:0099024	Plasma membrane invagination	26/337	127/18670	5.10E−18	4.79E−18	IGHV4-31/IGLL1/IGHV4-34/IGHM/IGHG4/IGHV3-43/IGHV3-53/IGHV3-72/IGHV3-66/IGHV4-59/IGHV3-20/IGHV3-23/IGHV3-73/IGHV3-13/IGHV2-26/IGHG3/IGHV2-70/IGHV1-69/IGLL5/IGHV3-30/IGHV3-64/IGKC/IGLC7/IGHV3-74/IGHG2/IGHV2-5	26
BP	GO:0002377	Immunoglobulin production	30/337	193/18670	2.21E−17	2.08E−17	IGLV5-45/IL13RA2/IGLV8-61/IGKV3D-15/IGLV1-44/IGKV2-30/IGKV5-2/IGLV2-8/IGLV6-57/IGKV1-27/IGLV3-21/IGLV1-36/IGLV2-14/IGLV4-69/IGLV3-16/IGKV1-5/IGKV1D-43/TRDV1/IGLV3-19/IGLV7-43/IGKV1D-13/IGKV1-17/IGKV1D-12/TRAV19/IGKV6D-21/IGKC/TRAV14DV4/CD22/IGLV7-46/IGLV4-60	30
BP	GO:0010324	Membrane invagination	26/337	135/18670	2.34E−17	2.20E−17	IGHV4-31/IGLL1/IGHV4-34/IGHM/IGHG4/IGHV3-43/IGHV3-53/IGHV3-72/IGHV3-66/IGHV4-59/IGHV3-20/IGHV3-23/IGHV3-73/IGHV3-13/IGHV2-26/IGHG3/IGHV2-70/IGHV1-69/IGLL5/IGHV3-30/IGHV3-64/IGKC/IGLC7/IGHV3-74/IGHG2/IGHV2-5	26
BP	GO:0002433	Immune response-regulating cell surface receptor signaling pathway involved in phagocytosis	26/337	139/18670	4.58E−17	4.31E−17	IGHV4-34/IGLV1-44/IGKV2-30/IGKV5-2/IGHG4/IGLV2-8/IGLV6-57/IGHV3-53/IGLV3-21/IGHV4-59/IGHV3-23/IGLV2-14/IGHV3-13/IGKV1-5/IGHG3/IGHV2-70/IGHV1-69/IGLV3-19/IGLV7-43/IGHV3-30/IGKV1-17/IGKV1D-12/IGKC/IGLC7/IGHG2/IGHV2-5	26
BP	GO:0038096	Fc-gamma receptor signaling pathway involved in phagocytosis	26/337	139/18670	4.58E−17	4.31E−17	IGHV4-34/IGLV1-44/IGKV2-30/IGKV5-2/IGHG4/IGLV2-8/IGLV6-57/IGHV3-53/IGLV3-21/IGHV4-59/IGHV3-23/IGLV2-14/IGHV3-13/IGKV1-5/IGHG3/IGHV2-70/IGHV1-69/IGLV3-19/IGLV7-43/IGHV3-30/IGKV1-17/IGKV1D-12/IGKC/IGLC7/IGHG2/IGHV2-5	26
BP	GO:0038094	Fc-gamma receptor signaling pathway	26/337	142/18670	7.34E−17	6.90E−17	IGHV4-34/IGLV1-44/IGKV2-30/IGKV5-2/IGHG4/IGLV2-8/IGLV6-57/IGHV3-53/IGLV3-21/IGHV4-59/IGHV3-23/IGLV2-14/IGHV3-13/IGKV1-5/IGHG3/IGHV2-70/IGHV1-69/IGLV3-19/IGLV7-43/IGHV3-30/IGKV1-17/IGKV1D-12/IGKC/IGLC7/IGHG2/IGHV2-5	26
BP	GO:0050871	Positive regulation of B cell activation	26/337	142/18670	7.34E−17	6.90E−17	IGHV4-31/IGLL1/IGHV4-34/IGHM/IGHG4/IGHV3-43/IGHV3-53/IGHV3-72/IGHV3-66/IGHV4-59/IGHV3-20/IGHV3-23/IGHV3-73/IGHV3-13/IGHV2-26/IGHG3/IGHV2-70/IGHV1-69/IGLL5/IGHV3-30/IGHV3-64/IGKC/IGLC7/IGHV3-74/IGHG2/IGHV2-5	26
BP	GO:0002431	Fc receptor mediated stimulatory signaling pathway	26/337	145/18670	1.22E−16	1.14E−16	IGHV4-34/IGLV1-44/IGKV2-30/IGKV5-2/IGHG4/IGLV2-8/IGLV6-57/IGHV3-53/IGLV3-21/IGHV4-59/IGHV3-23/IGLV2-14/IGHV3-13/IGKV1-5/IGHG3/IGHV2-70/IGHV1-69/IGLV3-19/IGLV7-43/IGHV3-30/IGKV1-17/IGKV1D-12/IGKC/IGLC7/IGHG2/IGHV2-5	26
BP	GO:0070613	Regulation of protein processing	28/337	180/18670	2.69E−16	2.52E−16	IGHV4-34/IGLV1-44/IGKV2-30/IGKV5-2/IGHG4/IGLV2-8/IGLV6-57/IGHV3-53/SPON1/IGLV3-21/IGHV4-59/IGHV3-23/IGLV2-14/IGHV3-13/CCBE1/IGKV1-5/IGHG3/IGHV2-70/IGHV1-69/IGLV3-19/IGLV7-43/IGHV3-30/IGKV1-17/IGKV1D-12/IGKC/IGLC7/IGHG2/IGHV2-5	28
BP	GO:0002440	Production of molecular mediator of immune response	34/337	286/18670	3.04E−16	2.86E−16	IGLV5-45/IL13RA2/IGLV8-61/IGKV3D-15/IGLV1-44/IGKV2-30/IGKV5-2/IGLV2-8/APOA1/IGLV6-57/IGKV1-27/IGLV3-21/IGLV1-36/KLK7/IGLV2-14/IGLV4-69/KLK3/IGLV3-16/IGKV1-5/IGKV1D-43/TRDV1/IGLV3-19/IGLV7-43/IGKV1D-13/KLK5/IGKV1-17/IGKV1D-12/TRAV19/IGKV6D-21/IGKC/TRAV14DV4/CD22/IGLV7-46/IGLV4-60	34
BP	GO:1903317	Regulation of protein maturation	28/337	182/18670	3.37E−16	3.16E−16	IGHV4-34/IGLV1-44/IGKV2-30/IGKV5-2/IGHG4/IGLV2-8/IGLV6-57/IGHV3-53/SPON1/IGLV3-21/IGHV4-59/IGHV3-23/IGLV2-14/IGHV3-13/CCBE1/IGKV1-5/IGHG3/IGHV2-70/IGHV1-69/IGLV3-19/IGLV7-43/IGHV3-30/IGKV1-17/IGKV1D-12/IGKC/IGLC7/IGHG2/IGHV2-5	28
BP	GO:0002673	Regulation of acute inflammatory response	26/337	159/18670	1.14E−15	1.07E−15	IGHV4-34/IGLV1-44/IGKV2-30/IGKV5-2/IGHG4/IGLV2-8/IGLV6-57/IGHV3-53/IGLV3-21/IGHV4-59/IGHV3-23/IGLV2-14/IGHV3-13/IGKV1-5/IGHG3/IGHV2-70/IGHV1-69/IGLV3-19/IGLV7-43/IGHV3-30/IGKV1-17/IGKV1D-12/IGKC/IGLC7/IGHG2/IGHV2-5	26
BP	GO:0002429	Immune response-activating cell surface receptor signaling pathway	42/337	473/18670	1.29E−15	1.22E−15	IGHV4-31/IGLL1/IGHV4-34/IGLV1-44/IGHM/IGKV2-30/IGKV5-2/IGHG4/IGLV2-8/IGHV3-43/IGLV6-57/IGHV3-53/IGHV3-72/IGHV3-66/IGLV3-21/IGHV4-59/IGHV3-20/IGHV3-23/IGHV3-73/IGLV2-14/IGHV3-13/IGKV1-5/IGHV2-26/IGHG3/IGHV2-70/IGHV1-69/IGLV3-19/IGLL5/MUCL1/TRBV7-9/IGLV7-43/IGHV3-30/IGHV3-64/IGKV1-17/IGKV1D-12/TRAV19/IGKC/IGLC7/CD22/IGHV3-74/IGHG2/IGHV2-5	42
BP	GO:0016485	Protein processing	35/337	328/18670	2.60E−15	2.44E−15	IGHV4-34/IGLV1-44/IGKV2-30/IGKV5-2/IGHG4/IGLV2-8/IGLV6-57/IGHV3-53/SPON1/KLK6/IGLV3-21/IGHV4-59/IGHV3-23/PCSK1N/IGLV2-14/IGHV3-13/KLK3/CCBE1/IGKV1-5/IGHG3/IGHV2-70/IGHV1-69/IGLV3-19/IGLV7-43/KLK1/IGHV3-30/PCSK9/SCG5/IGKV1-17/IGKV1D-12/IGKC/IGLC7/CPXM2/IGHG2/IGHV2-5	35
BP	GO:0050864	Regulation of B cell activation	27/337	184/18670	4.15E−15	3.90E−15	IGHV4-31/IGLL1/IGHV4-34/IGHM/IGHG4/IGHV3-43/IGHV3-53/IGHV3-72/IGHV3-66/IGHV4-59/IGHV3-20/IGHV3-23/IGHV3-73/IGHV3-13/IGHV2-26/IGHG3/IGHV2-70/IGHV1-69/IGLL5/IGHV3-30/IGHV3-64/IGKC/IGLC7/CD22/IGHV3-74/IGHG2/IGHV2-5	27
BP	GO:0008037	Cell recognition	27/337	215/18670	2.20E−13	2.07E−13	IGHV4-31/IGLL1/IGHV4-34/IGHM/IGHG4/IGHV3-43/IGHV3-53/IGHV3-72/CRTAC1/IGHV3-66/IGHV4-59/IGHV3-20/IGHV3-23/IGHV3-73/IGHV3-13/IGHV2-26/IGHG3/IGHV2-70/IGHV1-69/IGLL5/IGHV3-30/IGHV3-64/IGKC/IGLC7/IGHV3-74/IGHG2/IGHV2-5	27
BP	GO:0002526	Acute inflammatory response	27/337	220/18670	3.81E−13	3.58E−13	IGHV4-34/IGLV1-44/IGKV2-30/IGKV5-2/IGHG4/IGLV2-8/IGLV6-57/IGHV3-53/IGLV3-21/IGHV4-59/IGHV3-23/IGLV2-14/IGHV3-13/IGKV1-5/IGHG3/IGHV2-70/IGHV1-69/IGLV3-19/IGLV7-43/IGHV3-30/IGKV1-17/IGKV1D-12/IGKC/IL1A/IGLC7/IGHG2/IGHV2-5	27
BP	GO:0051604	Protein maturation	35/337	397/18670	8.46E−13	7.95E−13	IGHV4-34/IGLV1-44/IGKV2-30/IGKV5-2/IGHG4/IGLV2-8/IGLV6-57/IGHV3-53/SPON1/KLK6/IGLV3-21/IGHV4-59/IGHV3-23/PCSK1N/IGLV2-14/IGHV3-13/KLK3/CCBE1/IGKV1-5/IGHG3/IGHV2-70/IGHV1-69/IGLV3-19/IGLV7-43/KLK1/IGHV3-30/PCSK9/SCG5/IGKV1-17/IGKV1D-12/IGKC/IGLC7/CPXM2/IGHG2/IGHV2-5	35
BP	GO:0038095	Fc-epsilon receptor signaling pathway	23/337	169/18670	4.43E−12	4.16E−12	IGHV4-34/IGLV1-44/IGKV2-30/IGKV5-2/IGLV2-8/IGLV6-57/IGHV3-53/IGLV3-21/IGHV4-59/IGHV3-23/IGLV2-14/IGHV3-13/IGKV1-5/IGHV2-70/IGHV1-69/IGLV3-19/IGLV7-43/IGHV3-30/IGKV1-17/IGKV1D-12/IGKC/IGLC7/IGHV2-5	23
BP	GO:0042742	Defense response to bacterium	31/337	330/18670	5.32E−12	5.00E−12	IGHV4-31/IGLL1/IGHV4-34/IGHM/IGHG4/IGHV3-43/IGHV3-53/IGHV3-72/IGHV3-66/IGHV4-59/IGHV3-20/IGHV3-23/KLK7/IGHV3-73/IGHV3-13/KLK3/IGHV2-26/IGHG3/IGHV2-70/IGHV1-69/CAMP/IGLL5/IGHV3-30/KLK5/IGHV3-64/IGKC/IGLC7/IGHV3-74/CXCL13/IGHG2/IGHV2-5	31
BP	GO:0051251	Positive regulation of lymphocyte activation	31/337	334/18670	7.18E−12	6.74E−12	IGHV4-31/IGLL1/IGHV4-34/IGHM/SIRPG/IGHG4/IGHV3-43/IGHV3-53/IGHV3-72/CCL5/TNFSF9/IGHV3-66/IGHV4-59/IGHV3-20/IGHV3-23/IGHV3-73/IGHV3-13/IFNG/IGHV2-26/IGHG3/IGHV2-70/IGHV1-69/IGLL5/IGHV3-30/IGHV3-64/IGKC/IGF2/IGLC7/IGHV3-74/IGHG2/IGHV2-5	31
BP	GO:0038093	Fc receptor signaling pathway	26/337	241/18670	2.36E−11	2.22E−11	IGHV4-34/IGLV1-44/IGKV2-30/IGKV5-2/IGHG4/IGLV2-8/IGLV6-57/IGHV3-53/IGLV3-21/IGHV4-59/IGHV3-23/IGLV2-14/IGHV3-13/IGKV1-5/IGHG3/IGHV2-70/IGHV1-69/IGLV3-19/IGLV7-43/IGHV3-30/IGKV1-17/IGKV1D-12/IGKC/IGLC7/IGHG2/IGHV2-5	26
BP	GO:0051249	Regulation of lymphocyte activation	36/337	485/18670	5.50E−11	5.17E−11	IGHV4-31/IGLL1/IGHV4-34/IGHM/SOX11/SIRPG/LAG3/IGHG4/FOXN1/IGHV3-43/IGHV3-53/TIGIT/IGHV3-72/CCL5/TNFSF9/IGHV3-66/IGHV4-59/IGHV3-20/IGHV3-23/IGHV3-73/IGHV3-13/IFNG/IGHV2-26/IGHG3/IGHV2-70/IGHV1-69/IGLL5/IGHV3-30/IGHV3-64/IGKC/IGF2/IGLC7/CD22/IGHV3-74/IGHG2/IGHV2-5	36
BP	GO:0050851	Antigen receptor-mediated signaling pathway	29/337	316/18670	5.80E−11	5.45E−11	IGHV4-31/IGLL1/IGHV4-34/IGHM/IGHG4/IGHV3-43/IGHV3-53/IGHV3-72/IGHV3-66/IGHV4-59/IGHV3-20/IGHV3-23/IGHV3-73/IGHV3-13/IGHV2-26/IGHG3/IGHV2-70/IGHV1-69/IGLL5/TRBV7-9/IGHV3-30/IGHV3-64/TRAV19/IGKC/IGLC7/CD22/IGHV3-74/IGHG2/IGHV2-5	29
BP	GO:0002696	Positive regulation of leukocyte activation	31/337	380/18670	2.01E−10	1.89E−10	IGHV4-31/IGLL1/IGHV4-34/IGHM/SIRPG/IGHG4/IGHV3-43/IGHV3-53/IGHV3-72/CCL5/TNFSF9/IGHV3-66/IGHV4-59/IGHV3-20/IGHV3-23/IGHV3-73/IGHV3-13/IFNG/IGHV2-26/IGHG3/IGHV2-70/IGHV1-69/IGLL5/IGHV3-30/IGHV3-64/IGKC/IGF2/IGLC7/IGHV3-74/IGHG2/IGHV2-5	31
BP	GO:0002697	Regulation of immune effector process	34/337	458/18670	2.32E−10	2.18E−10	IL13RA2/IGHV4-34/IGLV1-44/IGKV2-30/IGKV5-2/ULBP2/LAG3/IGHG4/IGLV2-8/APOA1/IGLV6-57/IGHV3-53/IGLV3-21/IGHV4-59/IGHV3-23/KLK7/IGLV2-14/IGHV3-13/IFNG/IGKV1-5/IGHG3/IGHV2-70/IGHV1-69/IGLV3-19/IGLV7-43/IGHV3-30/KLK5/IGKV1-17/IGKV1D-12/IGKC/IGLC7/CD22/IGHG2/IGHV2-5	34
BP	GO:0050867	Positive regulation of cell activation	31/337	394/18670	4.90E−10	4.60E−10	IGHV4-31/IGLL1/IGHV4-34/IGHM/SIRPG/IGHG4/IGHV3-43/IGHV3-53/IGHV3-72/CCL5/TNFSF9/IGHV3-66/IGHV4-59/IGHV3-20/IGHV3-23/IGHV3-73/IGHV3-13/IFNG/IGHV2-26/IGHG3/IGHV2-70/IGHV1-69/IGLL5/IGHV3-30/IGHV3-64/IGKC/IGF2/IGLC7/IGHV3-74/IGHG2/IGHV2-5	31
BP	GO:0042113	B cell activation	27/337	310/18670	1.14E−09	1.07E−09	IGHV4-31/IGLL1/IGHV4-34/IGHM/IGHG4/IGHV3-43/IGHV3-53/IGHV3-72/IGHV3-66/IGHV4-59/IGHV3-20/IGHV3-23/IGHV3-73/IGHV3-13/IGHV2-26/IGHG3/IGHV2-70/IGHV1-69/IGLL5/IGHV3-30/IGHV3-64/IGKC/IGLC7/CD22/IGHV3-74/IGHG2/IGHV2-5	27
BP	GO:0006898	Receptor-mediated endocytosis	27/337	316/18670	1.74E−09	1.63E−09	APOB/IGHV4-34/IGLV1-44/IGKV2-30/IGKV5-2/IGLV2-8/APOA1/IGLV6-57/IGHV3-53/IGLV3-21/IGHV4-59/IGHV3-23/IGLV2-14/IGHV3-13/IGKV1-5/IGHV2-70/IGHV1-69/IGLV3-19/IGLV7-43/IGHV3-30/PCSK9/IGKV1-17/IGKV1D-12/IGKC/AMBP/IGLC7/IGHV2-5	27
BP	GO:0050900	Leukocyte migration	34/337	499/18670	2.23E−09	2.10E−09	IGLL1/APOB/IGHV4-34/IGLV1-44/IGHM/IGKV2-30/IGKV5-2/SIRPG/IGLV2-8/IGLV6-57/IGHV3-53/CCL5/IGLV3-21/IGHV4-59/IGHV3-23/IGLV2-14/IGHV3-13/IGKV1-5/IGHV2-70/IGHV1-69/IGLV3-19/MAG/SLC7A10/IGLV7-43/IGHV3-30/IGKV1-17/IGKV1D-12/IGKC/CXCL9/IGLC7/ATP1B2/EDN3/CXCL13/IGHV2-5	34
BP	GO:0050727	Regulation of inflammatory response	30/337	485/18670	3.25E−07	3.05E−07	IGHV4-34/IGLV1-44/IGKV2-30/IGKV5-2/IGHG4/IGLV2-8/APOA1/IGLV6-57/IGHV3-53/CCL5/GBP5/IGLV3-21/IGHV4-59/IGHV3-23/IGLV2-14/IGHV3-13/IGKV1-5/IGHG3/IGHV2-70/IGHV1-69/IGLV3-19/IGLV7-43/IGHV3-30/IGKV1-17/IGKV1D-12/IGKC/LRFN5/IGLC7/IGHG2/IGHV2-5	30
BP	GO:0042475	Odontogenesis of dentin-containing tooth	10/337	90/18670	0.000340129	0.000319658	WNT6/KLK4/SCN5A/FGF4/DLX3/BMP2/KLK5/CA2/FGF8/WNT10A	10
BP	GO:0031128	Developmental induction	6/337	34/18670	0.001891025	0.001777212	SIX3/WNT4/HOXC11/BMP2/FGF8/GDNF	6
BP	GO:0009952	Anterior/posterior pattern specification	14/337	219/18670	0.002987205	0.002807418	SIX3/HOXC8/HOXC6/ALX1/CRB2/FOXC2/HOXC10/HOXC11/BMP2/FGF8/HOXC9/CDX1/BARX1/NKD1	14
BP	GO:0021984	Adenohypophysis development	4/337	13/18670	0.004101352	0.003854509	WNT4/BMP2/FGF8/SOX2	4
BP	GO:0003002	Regionalization	18/337	351/18670	0.004611317	0.004333781	SIX3/HOXC8/HOXC6/ALX1/NKX2-2/CRB2/FOXC2/HOXC10/HOXC11/BMP2/FGF8/WNT7A/GDNF/HOXC9/CDX1/BARX1/DMRTA2/NKD1	18
BP	GO:0045165	Cell fate commitment	15/337	270/18670	0.007471561	0.00702188	WNT6/WNT4/FOXN1/NKX2-2/WNT16/FOXC2/HOXC10/BMP2/FGF8/TENM4/WNT7A/WNT5B/SOX2/WNT10A/DMRTA2	15
BP	GO:0098742	Cell–cell adhesion via plasma-membrane adhesion molecules	15/337	273/18670	0.008281927	0.007783474	PTPRT/APOA1/CLDN9/CRB2/CDH22/BMP2/MAG/LRRC4C/CLDN19/PCDH10/TENM4/CLDN6/TRO/PCDHB5/LRFN5	15
BP	GO:0042476	Odontogenesis	10/337	132/18670	0.008307838	0.007807825	WNT6/KLK4/SCN5A/FGF4/DLX3/BMP2/KLK5/CA2/FGF8/WNT10A	10
BP	GO:0061844	Antimicrobial humoral immune response mediated by antimicrobial peptide	7/337	73/18670	0.01961805	0.018437324	REG1A/KLK7/KLK3/CAMP/KLK5/CXCL9/CXCL13	7
BP	GO:0007389	Pattern specification process	19/337	446/18670	0.028662281	0.026937221	SIX3/HOXC8/WNT6/HOXC6/ALX1/NKX2-2/CRB2/FOXC2/HOXC10/HOXC11/BMP2/FGF8/WNT7A/GDNF/HOXC9/CDX1/BARX1/DMRTA2/NKD1	19
BP	GO:0001759	Organ induction	4/337	22/18670	0.032449794	0.03049678	HOXC11/BMP2/FGF8/GDNF	4
BP	GO:0048762	Mesenchymal cell differentiation	12/337	219/18670	0.035439505	0.033306553	ALX1/SOX11/WNT4/FGF19/CRB2/WNT16/FOXC2/BMP2/FGF8/GDNF/EDN3/WNT10A	12
BP	GO:0001658	Branching involved in ureteric bud morphogenesis	6/337	59/18670	0.035439505	0.033306553	WNT6/ADAMTS16/WNT4/BMP2/FGF8/GDNF	6
BP	GO:0016338	Calcium-independent cell-cell adhesion via plasma membrane cell-adhesion molecules	4/337	23/18670	0.036228036	0.034047626	CLDN9/BMP2/CLDN19/CLDN6	4
BP	GO:0045109	Intermediate filament organization	4/337	23/18670	0.036228036	0.034047626	TCHH/NEFH/GFAP/KRT71	4
BP	GO:0021871	Forebrain regionalization	4/337	24/18670	0.042179675	0.039641061	SIX3/BMP2/FGF8/DMRTA2	4
BP	GO:0046886	Positive regulation of hormone biosynthetic process	3/337	11/18670	0.042595197	0.040031574	WNT4/IFNG/PPARGC1A	3
BP	GO:0048864	Stem cell development	7/337	85/18670	0.042595197	0.040031574	ALX1/SOX11/FGF19/FOXC2/WNT7A/GDNF/EDN3	7
BP	GO:0021983	Pituitary gland development	5/337	42/18670	0.044221556	0.04156005	SIX3/WNT4/BMP2/FGF8/SOX2	5
BP	GO:0070268	Cornification	8/337	112/18670	0.04586542	0.043104977	TCHH/FLG/KRT71/KRT36/KRT5/KLK5/KRT31/KRT34	8
BP	GO:0008211	Glucocorticoid metabolic process	4/337	25/18670	0.04586542	0.043104977	WNT4/APOA1/BMP2/SERPINA6	4
CC	GO:0019814	Immunoglobulin complex	51/354	159/19717	1.95E−47	1.78E−47	IGHV4-31/IGLV5-45/IGLL1/IGLV8-61/IGHV4-34/IGKV3D-15/IGLV1-44/IGHM/IGKV2-30/IGKV5-2/IGHG4/IGLV2-8/IGHV3-43/IGLV6-57/IGHV3-53/IGHV3-72/IGHJ1/IGKV1-27/IGHV3-66/IGLV3-21/IGHV4-59/IGHV3-20/IGLV1-36/IGHV3-23/IGHV3-73/IGLV2-14/IGLV4-69/IGHV3-13/IGLV3-16/IGKV1-5/IGKV1D-43/IGHV2-26/IGHG3/IGHV2-70/IGHV1-69/IGLV3-19/IGLL5/IGLV7-43/IGKV1D-13/IGHV3-30/IGHV3-64/IGKV1-17/IGKV1D-12/IGKV6D-21/IGKC/IGLC7/IGHV3-74/IGLV7-46/IGLV4-60/IGHG2/IGHV2-5	51
CC	GO:0042571	Immunoglobulin complex, circulating	26/354	72/19717	3.37E−-25	3.07E−25	IGHV4-31/IGLL1/IGHV4-34/IGHM/IGHG4/IGHV3-43/IGHV3-53/IGHV3-72/IGHV3-66/IGHV4-59/IGHV3-20/IGHV3-23/IGHV3-73/IGHV3-13/IGHV2-26/IGHG3/IGHV2-70/IGHV1-69/IGLL5/IGHV3-30/IGHV3-64/IGKC/IGLC7/IGHV3-74/IGHG2/IGHV2-5	26
CC	GO:0009897	External side of plasma membrane	39/354	393/19717	3.99E−16	3.63E−16	IGHV4-31/IGLL1/IL13RA2/IGHV4-34/IGHM/CNTFR/ULBP2/CCR10/LAG3/LY6G6C/IGHG4/IGHV3-43/GPIHBP1/IGHV3-53/IGHV3-72/CD8A/IGHV3-66/IGHV4-59/IGHV3-20/IGHV3-23/IGHV3-73/GFRA2/IGHV3-13/GFRA1/IGHV2-26/IGHG3/IGHV2-70/IGHV1-69/IGLL5/IGHV3-30/PCSK9/IGHV3-64/FGF8/IGKC/CXCL9/IGLC7/IGHV3-74/IGHG2/IGHV2-5	39
CC	GO:0072562	Blood microparticle	15/354	147/19717	5.34E−06	4.86E−06	IGHM/IGKV2-30/IGHG4/APOA1/IGLV3-21/IGHV3-23/IGHV3-13/IGKV1-5/IGHG3/IGKV1-17/IGKV1D-12/IGKC/AMBP/PON1/IGHG2	15
CC	GO:0042101	T cell receptor complex	11/354	127/19717	0.001186365	0.001080471	TRBV7-6/CD8A/TRBV4-2/TRAV21/TRDV1/TRBV7-9/TRAV19/TRAV12-1/TRAV14DV4/TRBV4-1/TRAV26-2	11
CC	GO:0098802	Plasma membrane receptor complex	15/354	295/19717	0.016378041	0.01491615	GRIK5/CNTFR/TRBV7-6/CD8A/TRBV4-2/TRAV21/TRDV1/BMP2/TRBV7-9/CHRNA3/TRAV19/TRAV12-1/TRAV14DV4/TRBV4-1/TRAV26-2	15
MF	GO:0003823	Antigen binding	41/300	160/17697	3.33E−34	2.97E−34	IGHV4-31/IGLL1/IGHV4-34/IGLV1-44/IGHM/IGKV2-30/IGKV5-2/LAG3/IGHG4/IGLV2-8/IGHV3-43/IGLV6-57/IGHV3-53/IGHV3-72/IGHV3-66/IGLV3-21/IGHV4-59/IGHV3-20/IGHV3-23/IGHV3-73/IGLV2-14/IGHV3-13/IGKV1-5/IGHV2-26/IGHG3/IGHV2-70/IGHV1-69/IGLV3-19/IGLL5/TRBV7-9/IGLV7-43/IGHV3-30/IGHV3-64/IGKV1-17/IGKV1D-12/TRAV19/IGKC/IGLC7/IGHV3-74/IGHG2/IGHV2-5	41
MF	GO:0034987	Immunoglobulin receptor binding	26/300	76/17697	5.43E−25	4.85E−25	IGHV4-31/IGLL1/IGHV4-34/IGHM/IGHG4/IGHV3-43/IGHV3-53/IGHV3-72/IGHV3-66/IGHV4-59/IGHV3-20/IGHV3-23/IGHV3-73/IGHV3-13/IGHV2-26/IGHG3/IGHV2-70/IGHV1-69/IGLL5/IGHV3-30/IGHV3-64/IGKC/IGLC7/IGHV3-74/IGHG2/IGHV2-5	26
MF	GO:0005109	Frizzled binding	8/300	39/17697	3.98E−05	3.55E−05	WNT6/MYOC/WNT4/RSPO4/WNT16/WNT7A/WNT5B/WNT10A	8
MF	GO:0048018	Receptor ligand activity	23/300	482/17697	0.001065684	0.000951118	WNT4/FGF19/APOA1/REG1A/CCL5/TNFSF9/FGF4/INSL4/SLURP1/IFNG/TRH/FGF20/BMP2/FGF8/CXCL9/WNT7A/GDNF/IL1A/FGF3/IGF2/EDN3/WNT10A/CXCL13	23
MF	GO:0005104	Fibroblast growth factor receptor binding	5/300	25/17697	0.00536912	0.004791912	FGF19/FGF4/FGF20/FGF8/FGF3	5
MF	GO:0004252	Serine-type endopeptidase activity	10/300	160/17697	0.033673709	0.030053615	KLK4/KLK6/CFD/KLK7/KLK3/GZMH/KLK8/KLK1/KLK5/PCSK9	10
MF	GO:0046873	Metal ion transmembrane transporter activity	18/300	438/17697	0.036391745	0.032479448	GRIK5/TTYH1/GPM6A/CACNA1S/SCN5A/KCNE5/LRRC55/KCNJ15/SLC5A8/JPH3/CACNG6/CACNA1I/ATP2A3/SLC6A15/SLC13A5/SCN1A/SLC13A2/ATP1B2	18
MF	GO:0022839	Ion gated channel activity	15/300	334/17697	0.037581202	0.033541033	GRIK5/TTYH1/CACNA1S/SCN5A/ANO2/KCNE5/LRRC55/KCNJ15/P2RX2/GABRA3/JPH3/CACNG6/CACNA1I/CHRNA3/SCN1A	15
MF	GO:0022836	Gated channel activity	15/300	343/17697	0.043831647	0.039119522	GRIK5/TTYH1/CACNA1S/SCN5A/ANO2/KCNE5/LRRC55/KCNJ15/P2RX2/GABRA3/JPH3/CACNG6/CACNA1I/CHRNA3/SCN1A	15
MF	GO:0070325	Lipoprotein particle receptor binding	4/300	26/17697	0.044441069	0.039663429	APOB/APOA1/PCSK9/SYT1	4
MF	GO:0008236	Serine-type peptidase activity	10/300	182/17697	0.049388397	0.044078894	KLK4/KLK6/CFD/KLK7/KLK3/GZMH/KLK8/KLK1/KLK5/PCSK9	10
MF	GO:0005343	Organic acid:sodium symporter activity	4/300	28/17697	0.049388397	0.044078894	SLC5A8/SLC6A15/SLC13A5/SLC13A2	4
MF	GO:0017171	Serine hydrolase activity	10/300	186/17697	0.049706953	0.044363203	KLK4/KLK6/CFD/KLK7/KLK3/GZMH/KLK8/KLK1/KLK5/PCSK9	10

**Fig. 7:**
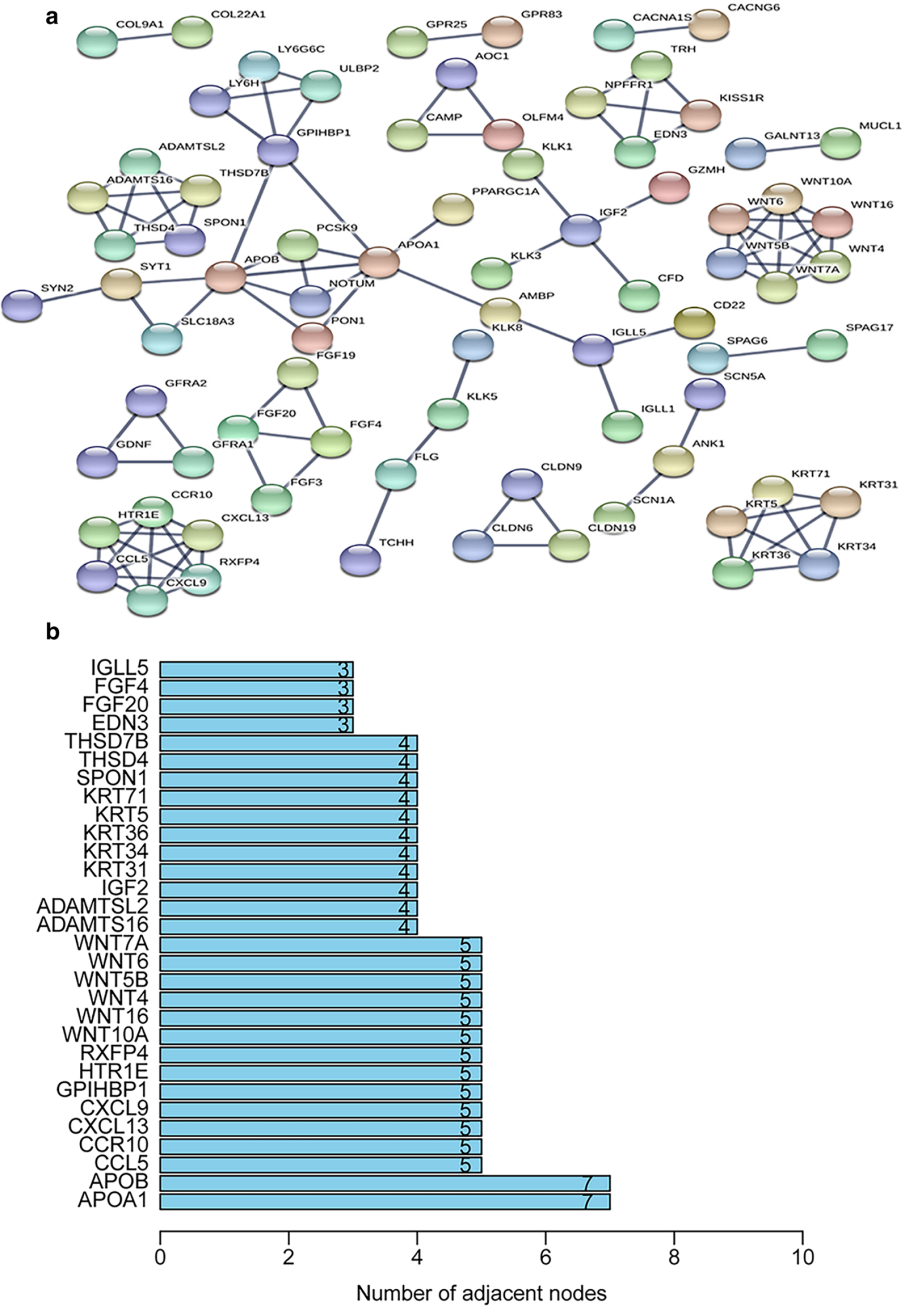
Protein interactions between the DEGs. **a** PPI network of the DEGs. **b** Histogram of the number of adjacent nodes

Regarding molecular functions, most genes were enriched for antigen binding, immunoglobulin receptor binding, and receptor-ligand activity. In the KEGG pathway enrichment analysis of the DEGs, these genes showed notable associations with breast cancer, basal cell carcinoma, gastric cancer, hippo signaling pathway, and the Wnt signaling pathway (Fig. 6b and Table 3). String was utilized to confirm the relationship between DEGs and then construct networks describing their interactions (Fig. 7a). The most common neighbors in the PPI diagram were APOA1 and APOB (Fig. 7b).

**Table 3:** The top KEGG exhaustive items of the DEGs in UCEC

ID	Description	GeneRatio	BgRatio	p.adjust	qvalue	Count
hsa05224	Breast cancer	11/115	147/8058	0.000788178	0.000687802	11
hsa05226	Gastric cancer	11/115	149/8058	0.000788178	0.000687802	11
hsa04390	Hippo signaling pathway	11/115	157/8058	0.000865824	0.00075556	11
hsa04310	Wnt signaling pathway	10/115	160/8058	0.003453597	0.003013774	10
hsa05217	Basal cell carcinoma	7/115	63/8058	0.001385335	0.00120891	7

### Association between differential immune gene expressions and immune infiltration

**Fig. 8:**
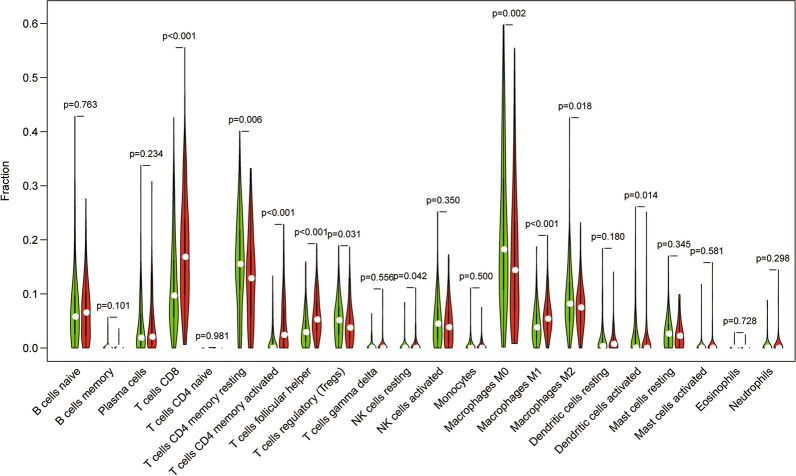
The violin diagram depicts the expression analysis of multiple immune cells in the high and low TMB groups. The green color is used for the high TMB group, and the red color is used for the low TMB group

**Fig. 9:**
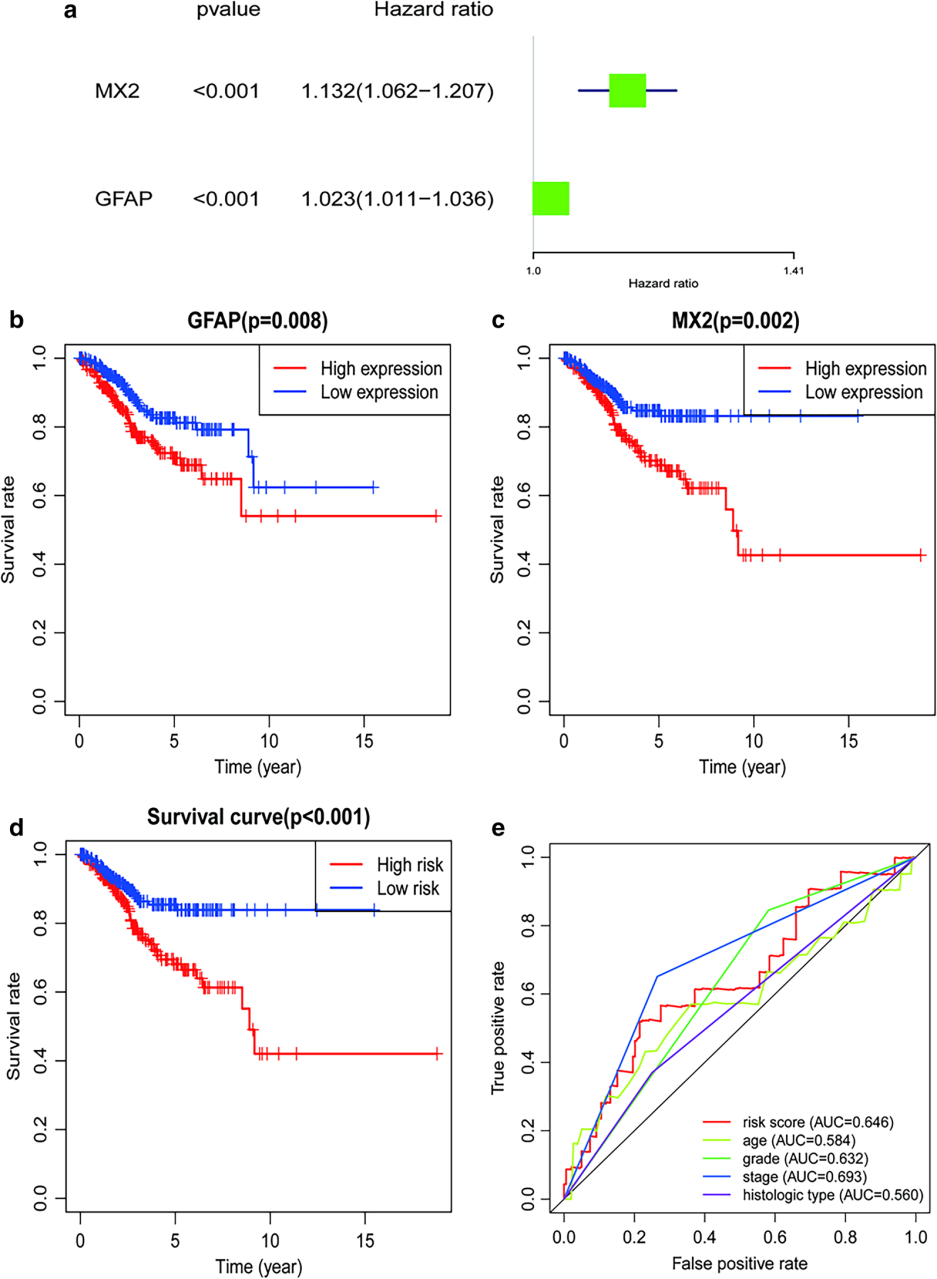
Differential immune genes in OS. **a** Forest plot of the differential immune genes; **b**, **c** survival analysis of the GFAP and MX2 genes; **d **survival analysis of the risk score; **e** the ROC curves of the models

**Fig. 10:**
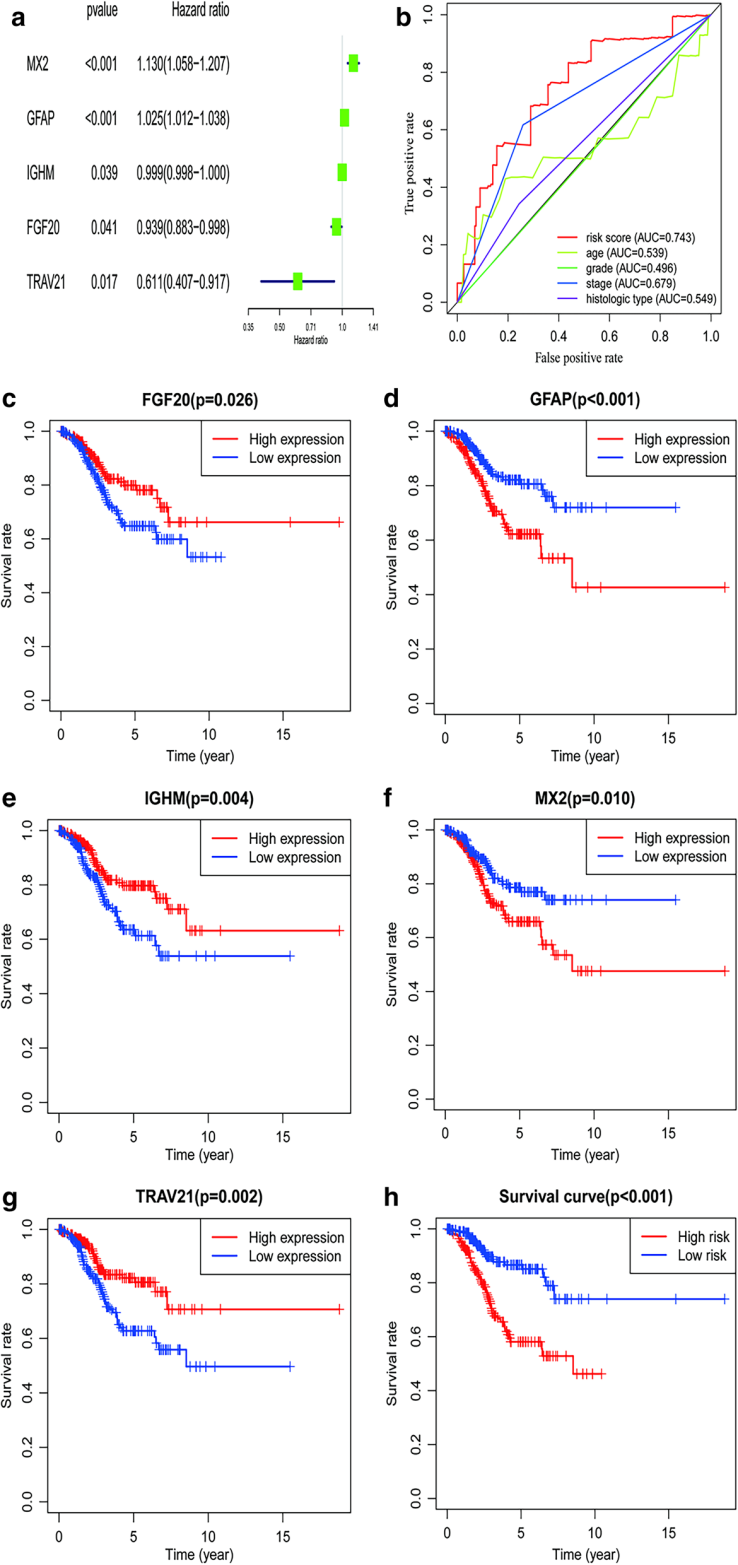
Differential immune genes in DFS. **a** Forest plot of the differential immune genes; **b** the ROC curves of the models; **c**–**g** Survival analysis of GFAP, MX2, FGF20, IGHM, and TRAV21 genes; **h** survival analysis of the risk score

The violin plot shows the difference between various immune cell types in the high and low TMB groups. As suggested by the results, T cells CD8 (P < 0.001), T cells CD4 memory resting (P = 0.006), T cells CD4 memory activated (P < 0.001), T cells follicular helper (P < 0.001), T cells regulatory (P = 0.031), NK cells resting (P = 0.042), Macrophages M0 (P = 0.002), Macrophages M1 (P < 0.001), Macrophages M2 (P = 0.018) and Dendritic cells activated (P = 0.014) showed significant differences between the high and low TMB groups (Fig. 8). Differential immune genes showed significant associations between their expression patterns, and the OS and DFS for UCEC patients were used to conduct a univariate Cox regression analysis. Eventually, we found GFAP (HR 1.023, 95%CI 1.011,1.036, P < 0.001) and MX2 (HR 1.132, 95%CI 1.062,1.207, P < 0.001) to be related to overall survival after multivariate Cox regression analysis was used to remove genes that were not independent indicators of prognosis (Fig. 9a). It could be detected that high expression of GFAP and MX2 were related to poorer OS in endometrial cancer patients, based on the values of the long-rank test and Kaplan-Meier curve (Fig. 9b, c). MX2 (HR 1.130, 95%CI 1.058, 1.207, P < 0.001), GFAP (HR 1.025, 95%CI 1.012, 1.038, P < 0.001), IGHM (HR 0.999, 95%CI 0.998, 1.000, P = 0.039), FGF20 (HR 0.939, 95%CI 0.883, 0.998, P = 0.041) and TRAV21 (HR 0.611, 95%CI 0.407, 0.917, P = 0.017) were analyzed for DFS (Fig. 10a). Regarding DFS, UECE patients with a higher expression level of MX2 and GFAP had a worse prognosis, while patients with higher mRNA levels of IGHM, FGF20, and TRAV21 showed a favorable outcome (Fig. 8c–h). These genes and clinical factors, including age, grade, stage, histological type, which are related to prognosis, were selected for model construction. The prognosis index formula multiplied the gene expression level and clinical characteristics in each case with the corresponding Cox regression coefficient. All selected genes were classified into either a high-risk or low-risk group based on the median prognosis index value, and survival curves were plotted. Moreover, Kaplan-Meier plots were drawn to analyze differences in survival time between both groups, indicating that cases in the low-risk group were significantly associated with increased survival probability than those in the high-risk group, for both OS and DFS (Figs. 9d and 10h). As shown in Figs. 8e and 9b, the largest AUC areas were found for the risk score of OS (0.646) and DFS (0.743), respectively.

### Association between risk score and immune infiltrates

**Fig. 11:**
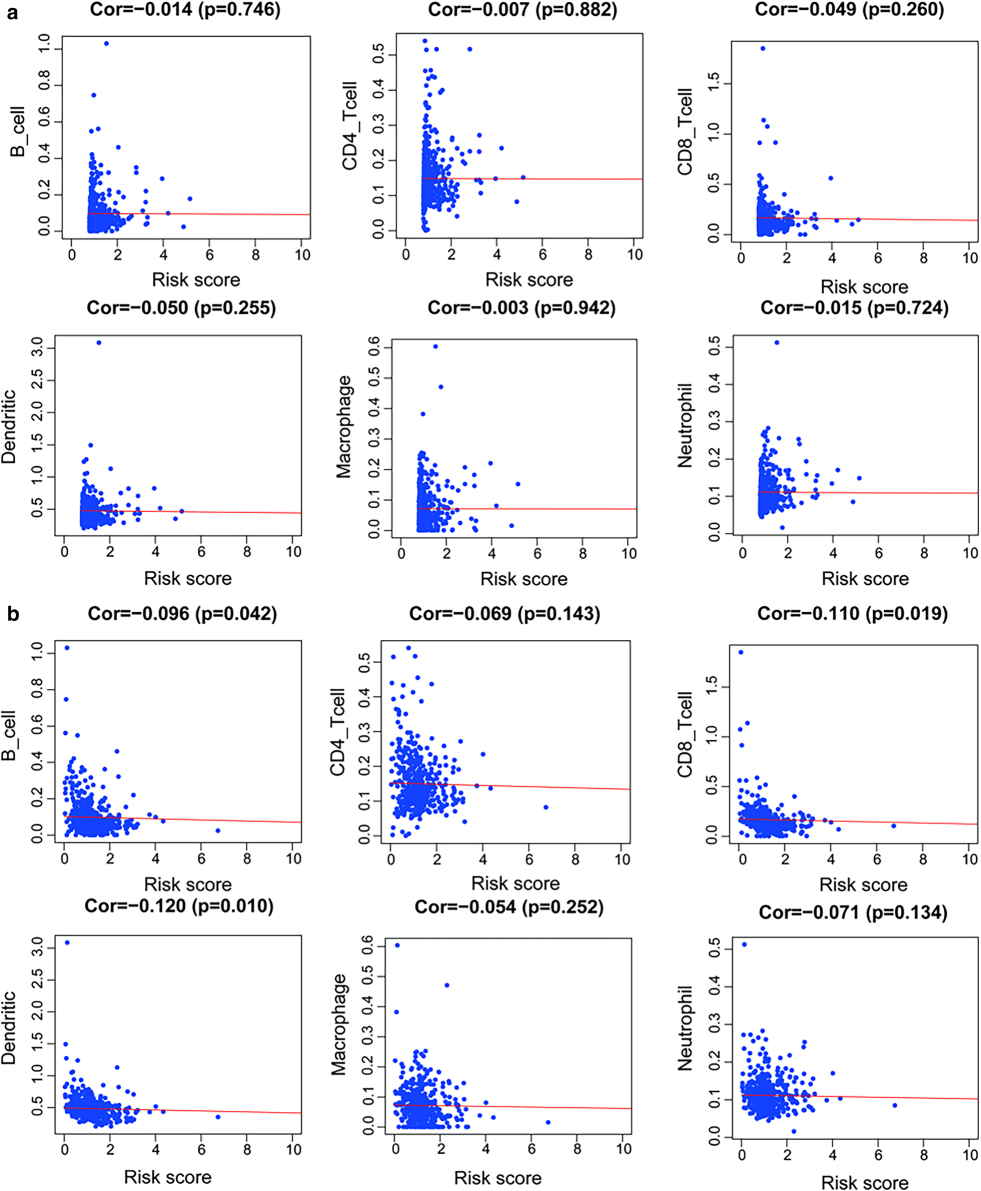
Correlation analysis of the risk score and immune cells in OS (**a**) and DFS (**b**)

The relationship between risk score and different types of immune cells were obtained from the Timer website using R software. The risk score of DFS showed a negative correlation with CD4+ T cell, CD8+ T cell, macrophage, and neutrophil (all P < 0.05, Fig. 11a). However, the risk score of OS was not associated with immune cells (all P < 0.05, Fig. 11b).

### High B cell and CD8 + T cell infiltration indicates a better outcome

**Fig. 12:**
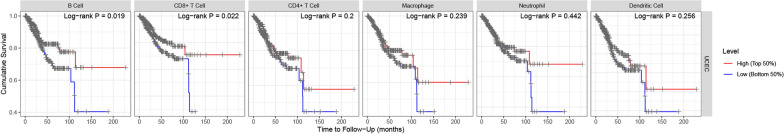
The Kaplan–Meier plot of the six immune cells: B cell, CD8+ T cell, CD4+ T cell, macrophage, neutrophil, and dendritic cell

To understand the association between immune infiltrating cells and survival in UCEC, we used the Cox regression equation to calculate the expression levels of CD4+ T cell (HR 0.001, 95%CI 0, 0.052, P = 0.005), CD8+ T cell (HR 0.001, 95%CI 0, 0.052, P = 0.001) and Neutrophil (HR 2314.933, 95%CI 1.836, 2919028.574, P = 0.033), which had declined due to cancer progression (Table 4). Additionally, Kaplan-Meier plots were applied to determine that UECE patients with a higher expression level of B cell and CD8+ T cell had a better prognosis (Fig. 12).

**Table 4:** Multi-factor analysis of six immune infiltrating cells in UCEC

	HR	95%CI_low	95%CI_up	p.value
B cell	0.102	0	47.135	0.466
CD8+ Tcell	0.001	0	0.052	0.001**
CD4+ Tcell	0	0	0.068	0.005**
Macrophage	24.031	0.178	3252.991	0.204
Neutrophil	2314.933	1.836	2919028.574	0.033*
Dendritic	4.928	0.183	132.438	0.342

*P < 0.05; **P < 0.01

### Nomogram of the differential immune genes and clinical variables for OS and DFS

**Fig. 13:**
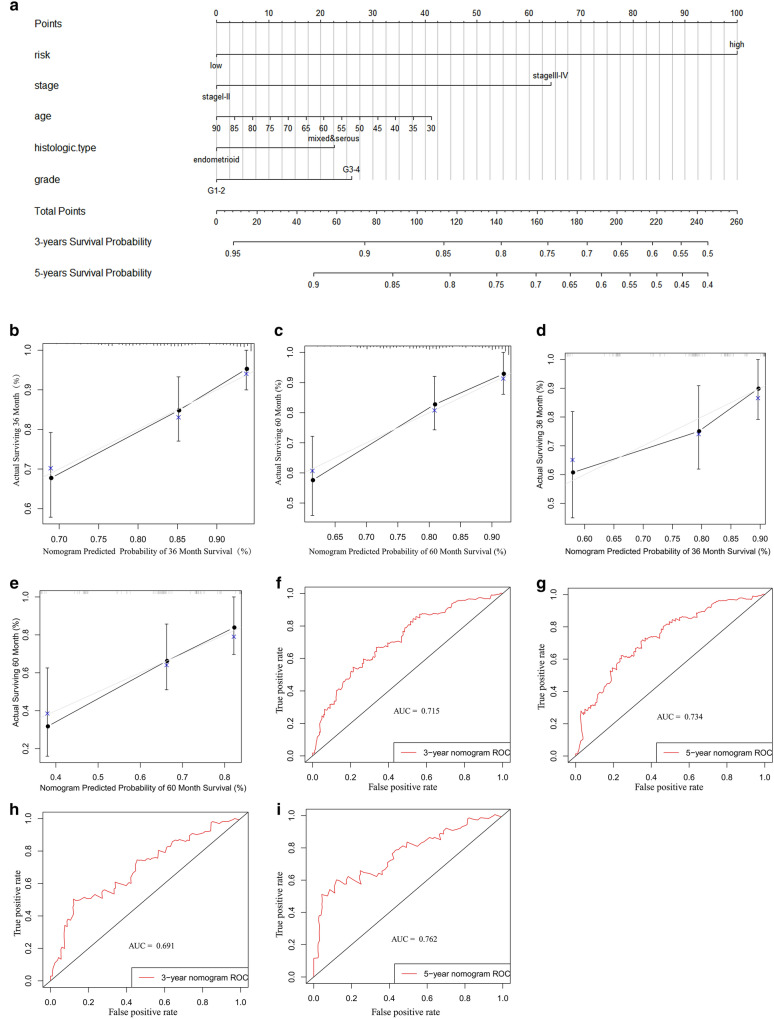
The model of risk score and clinical factors in OS. **a** 3-year and 5-year survival nomogram; **b**–**e** 3-year and 5-year calibration charts for the training and verification groups; **f**–**i** 3-year and 5-year ROC graphs for the training and verification groups

**Fig. 14:**
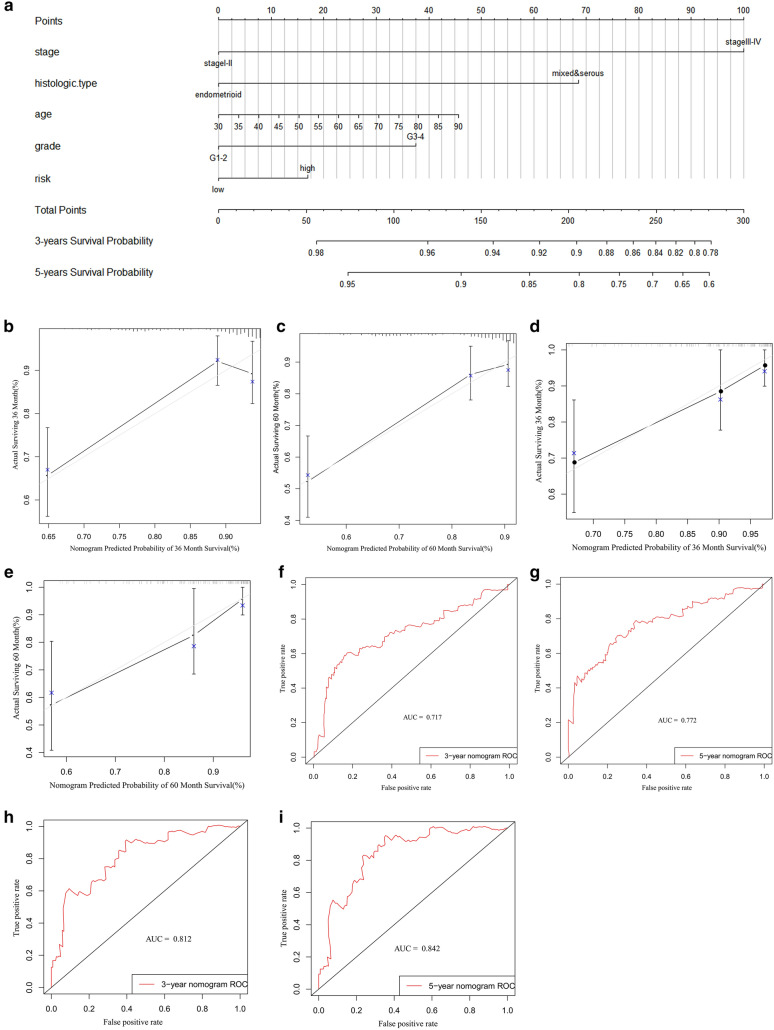
The model of risk score and clinical factors in DFS. **a** 3-year and 5-year survival nomogram; **b**–**e** 3-year and 5-year verification plots in testing and verification cohort; **f**–**i** 3-year and 5-year AUC areas in testing and verification cohort

The basic clinical characteristics of the patients are shown in Table 5 for OS and in Table 6 for DFS. Through univariate and multivariate Cox analyses of the modeling group and the entire cohort of patients, we found that the risk score (especially for DFS) may be an independent risk factor for UCEC patients (Tables 7, 8, 9, 10). Additionally, we used risk group, age, grade, stage, and histological type to construct nomogram models for OS (Fig. 13a) and DFS (Fig. 14a). To verify the predictive value of the models, 3-year and 5-year calibration charts were drawn for the modeling group and the verification group for OS (Fig. 13b–e) and DFS (Fig. 14b–e), which suggested the two models produced consistent results. ROC curves were constructed, and their AUC areas were greater than 0.7, indicating that these two models showed a high level of diagnostic accuracy.

**Table 5:** Clinical information of UCEC patients for OS obtained from TCGA database

Variables	UCEC
	N = 521(%)
Age	64(57,71)
Stage	
Stage I–II	374(71.8%)
Stage III–IV	147(28.2%)
Grade	
G1–2	212(40.7%)
G3–4	309(59.3%)
Histologic type	
Endometrioid	388(74.5%)
Mixed & serous	133(25.5%)
Risk	
Low	261(50.1%)
High	260(49.9%)

**Table 6:** Clinical baseline of UCEC patients for DFS obtained from TCGA database

Variables	UCEC
	N = 450(%)
Age	63(56.8,70)
Stage	
Stage I–II	328(72.9%)
Stage III–IV	122(27.1%)
Grade	
G1–2	195(43.3%)
G3–4	255(56.7%)
Histologic type	
Endometrioid	339(75.3%)
Mixed & serous	111(24.7%)
Risk	
Low	224(49.8%)
High	226(50.2%)

**Table 7:** Univariate and multivariate Cox analysis of UCEC patients for OS in the training set

Variables	Univariate analysis		Multivariable analysis	
	HR(95%CI)	P-value	HR(95%CI)	P-value
Age	1.020(0.995–1.046)	0.177	1.009(0.981–1.037)	0.546
Stage				
Stage I–II	Reference		Reference	
Stage III–IV	4.175(2.506–6.957)	< 0.001	3.045(1.769–5.241)	< 0.001
Grade				
G1–2	Reference		Reference	
G3–4	3.264(1.697–6.279)	< 0.001	1.518(0.707–3.261)	0.284
Histologic type				
Endometrioid	Reference		Reference	
Mixed & serous	3.939(2.374–6.537)	< 0.001	2.146(1.101–4.181)	0.025
Risk				
Low	Reference		Reference	
High	2.435(1.403–4.226)	0.002	1.209(0.628–2.325)	0.57

**Table 8:** Univariate and multivariate Cox analysis of all UCEC patients for OS

Variables	Univariate analysis		Multivariable analysis	
	HR(95%CI)	P-value	HR(95%CI)	P-value
Age	1.031(1.009–1.053)	0.005	1.023(1.000-1.046)	0.047
Stage				
Stage I–II	Reference		Reference	
Stage III–IV	3.819(2.471–5.901)	< 0.001	3.091(1.957–4.882)	< 0.001
Grade				
G1–2	Reference		Reference	
G3–4	3.589(2.018–6.382)	< 0.001	2.135(2.122–4.064)	0.021
Histologic type				
Endometrioid	Reference		Reference	
Mixed & serous	2.856(1.851–4.406)	< 0.001	1.241(0.734–2.097)	0.421
Risk				
Low	Reference		Reference	
High	2.166(1.368–3.431)	0.001	1.271(0.758–2.130)	0.363

**Table 9:** Univariate and multivariate Cox analysis of UCEC patients for DFS in the modeling group

Variables	Univariate analysis		Multivariable analysis	
	HR(95%CI)	P-value	HR(95%CI)	P-value
Age	1.005(0.980–1.031)	0.692	0.992(0.967–1.019)	0.572
Stage				
Stage I–II	Reference		Reference	
Stage III–IV	2.430(1.423–4.149)	0.001	2.026(1.143–3.593)	0.016
Grade				
G1–2	Reference		Reference	
G3–4	1.989(1.096–3.609)	0.024	1.330(0.655-2.700)	0.429
Histologic type				
Endometrioid	Reference		Reference	
Mixed & serous	2.082(1.213–3.573)	0.008	1.281(0.661–2.481)	0.463
Risk				
Low	Reference		Reference	
High	3.159(1.715–5.818)	0.002	3.000(1.606–5.606)	0.001

**Table 10:** Univariate and multivariate Cox analysis of all UCEC patients for DFS

Variables	Univariate analysis		Multivariable analysis	
	HR(95%CI)	P-value	HR(95%CI)	P-value
Age	1.009(0.989–1.030)	0.395	0.995(0.974–1.017)	0.676
Stage				
Stage I–II	Reference		Reference	
Stage III–IV	2.125(1.389–3.251)	0.001	1.746(1.101–2.769)	0.018
Grade				
G1–2	Reference		Reference	
G3–4	2.103(1.312–3.372)	0.002	1.478(0.852–2.564)	0.164
Histologic type				
Endometrioid	Reference		Reference	
Mixed & serous	2.212(1.437–3.404)	< 0.001	1.251(0.734–2.133)	0.411
Risk				
Low	Reference		Reference	
High	3.015(1.868–4.866)	0.001	2.799(1.707–4.589)	< 0.001

## Discussion

Immunotherapy has been used to treat recurring or metastatic tumors in clinical settings, but it is only effective for certain tumors, such as breast cancer [16] and prostate cancer [17]. In recent years, an increasing number of research studies have focused on immune molecular features and immunotherapeutic intervention for UCEC [18], owing to the lower survival rates of advanced UCEC patients. It was found that TMB can be beneficial to understand gene mutations in cancer cells, which is closely related to the efficacy of immunotherapy [19]. During the further analysis of the correlation between TMB and UCEC, we first explored the association between the high and low TMB groups with clinical survival. We found that for both OS and DFS, high TMB indicated a better prognosis. High TMB indicates that cancer cells have a high level of mutations, suggesting that cancer cells are more different from normal cells. They can be easily discovered by the human immune system, allowing the tumor cells to be killed.

The more effective the immunotherapy, the longer the survival [20, 21]. Of course, the detail mechanisms of that are unclear. A leading research demands that the process of mutations may create neoantigens which play an important role in the response of patients to immune checkpoint inhibitors. On the other hand, the expense of immunotherapy was still high, and many studies have paid an increasing amount of attention to determine whether patients were suitable for immunotherapy. TMB assessment can be used to predict the efficacy of immunotherapy further. Wu et al. [22] found that progression-free survival (PFS) (combined HR 0.59, 95%CI 0.49, 0.71, P < 0.001) and OS (combined HR 0.68, 95%CI 0.53, 0.89, P = 0.004) of the high TMB group significantly improved through the study of TMB levels and the effect of immunotherapy on various tumors. In the predictive analysis, TMB was found to be an independent predictor of immune checkpoint inhibitor treatment.

In contrast, group testing found that patients with high TMB showed an excellent response to immunotherapy [23], indicating that a higher TMB is expected to improve the efficacy of immune checkpoint inhibitors in cancers. Then, we used the CIBERSORT algorithm to calculate the proportion of immune cell infiltration in each patient sample. Additionally, the two TMB groups were strongly associated with specific immune infiltrating cells, which further proved that TMB was associated with the immune response. At the same time, high TMB was related to immunotherapy, and this phenomenon may involve immune infiltrating cells.

Using the median value of TMB as the critical value, we identified 393 DEGs between the low and high expression groups of TMB. Functional enrichment analyses of those DEGs provided an understanding of their biological roles. In the GO analysis, immune cells and receptors were found to be associated with the DEGs. Sonoda et al. [24] suggested that RCAS1 was a ligand for immune cell receptors, which was significantly associated with the stage of UCEC, the degree of myometrial invasion, positive peritoneal cytology, and overall survival. In the KEGG analysis, they were associated with various tumor pathways or genes, such as basal cell, breast, and gastric carcinoma. This implied that they had specific tumor pathways in common. For example, there were some similarities between UCEC and breast cancer at the molecular level. IDO1 was involved in the anti-tumor immune process of both tumors and was related to TMB [18]. After the protein network map of the DEGs was constructed, we detected the core expression levels of these genes in the PPI network, which may play an essential role in UCEC. APOA1 could promote the increase of macrophage infiltration, decrease TMB and metastasis, and improve the survival rate, similar to its effects in colorectal cancer [25, 26]. APOB caused a high mutation burden of cancer genes and tumorigenesis [27].

We utilized differential immune genes to establish Cox risk models for OS and DFS. Two genes (GFAP and MX2) were identified in the OS model, while five genes (GFAP, MX2, FGF20, IGHM, and TRAV21) were identified in the DFS model. In this study, we further explored the role of the above-mentioned differential immune genes and immune infiltration in the high and low TMB groups in UCEC. These genes were the core differential immune genes, and the risk scores obtained from the Cox multivariable analysis were grouped. For DFS, the risk score was related to some immune cells. The higher the risk score for DFS, the lower the level of CD4+ T cell, CD8+ T cell, macrophage, and neutrophil infiltration.

Additionally, through the survival analysis, we found that high-risk scores indicated better survival. MX2 is a viral interferon, which is the key to block human immunodeficiency virus type 1 [28]. GFAP can delay the development of type 1 diabetes by regulating T cell differentiation [29]. Inhibition of the FGFR family of genes can prevent the development of tumors by blocking paracrine signaling, which was related to immune escape in the tumor microenvironment [30].

We found that decreases in the expression of B cell and CD8+ T cell showed an apparent association with a poorer prognosis. In breast cancer, the presence of CD8+ T cells decreased the risk of breast cancer death by about 20% [31]. CD8+ T cells induced prolonged survival for patients with various types of tumors, including liver cancer [32] and rectal cancer [33]. Previous research [34] has proven that CD8+ T lymphocytes are an independent risk factor and that UCEC patients with high expression levels show better survival rates, which is consistent with our results. The role of B cells on tumors is unclear. B cells promote squamous cell carcinoma (SCC) development by depositing immunoglobulin- containing immune complexes [35]. In our study, high B cell expression was associated with a good outcome. Although the role of B cells in tumors is uncertain, the prognostic value of B-cell gene expression signatures in tumors has been demonstrated [36]. From the above the mechanisms that B cell and CD8+ T cell were linked to immunosuppression in tumor microenvironment are incompletely clear. Advanced research implied that these immunotherapy are potentially related to the expression of PD1 and CTLA4 from CD8+ T cell.

Finally, the risk stratification in the models mentioned above and other clinical factors was used to conduct single-factor and multifactor Cox analyses to construct the corresponding Nomogram model. The ROC curve showed that the Nomogram model was more reliable than the other models. The nomogram could comprehensively evaluate the survival rate of patients with genetic and clinical factors, and they were more intuitive and performed well. Furthermore, the risk score in DFS showed a significantly greater impact on diagnosis than in OS. At present, an increasing number of genes have been used as models to predict the prognosis and improve the prognosis of UCEC [37].

However, this study also contains certain limitations: (i) One limitation of this study is that it was a retrospective study. The relationship between differential TMB-related immune genes and immune cell infiltration still needs to be confirmed using primary experimental evidence. For example, we can analyse the differential immune cell infiltration expression between TMB^High^ and TMB^Low^ goup of EC cells and further explore the expressed level of immune checkpoint inhibitors such as PD-1 or CTL-4. (ii) The lack of many clinical samples to verify the prognostic effect of TMB and its potential relationship with immune infiltration. Therefore, the relationship between the occurrence and development of EC needs to be further confirmed using many more studies. Due to cost and technological limitations, the application of polygenic models is restricted.

## Conclusions

High TMB is related to prolonged survival and may promote immune infiltration. The immune gene models related to TMB levels were established. Clinical factors related to the models were determined to evaluate the prognosis of UCEC further and provide a base to predict immunotherapeutic outcomes. Modeling of immune infiltrating cells in endometrial cancer also showed that B cell and CD8+ T cell are the most important types of immune infiltrating cells. The function and mechanism of involvement of these hub immune genes, included in the models, are still unclear. Many more experiments need to be conducted to confirm their function in the immune system (Additional files 1, 2, 3, 4, 5, 6, 7).

## Supplementary Information

**Additional file 1.** The disease-free survival of six immune cells.

**Additional file 2.** Clinical features of uterine corpus endometrial carcinoma patients in disease-free survival.

**Additional file 3.** Related genes in immune cells.

**Additional file 4.** The overall survival of six immune cells.

**Additional file 5.** Clinical features of uterine corpus endometrial carcinoma patients in overall survival.

**Additional file 6.** The tumor mutation burden of uterine corpus endometrial carcinoma samples.

**Additional file 7.** The mRNA data of uterine corpus endometrial carcinoma samples.

## Data Availability

The datasets used and/or analysed during the current study are available from the corresponding author on reasonable request.

## Abbreviations

AUC: Area under the curve
CIs: Confidence interval
CTLA-4: Cytotoxic T-lymphocyte antigen 4
DEGs: Differentially expressed genes
DFS: Disease-free survival
GEP: Gene expression profiles
GO: Gene Ontology
HR: Hazard ratio
ICI: Immune Checkpoint Inhibitor
KEGG: Kyoto Encyclopedia of Genes, Genomes
OS: Overall survival
PD-1: Programed cell death 1 receptor
PFS: Progression-free survival
POLE: polymerase ɛ
PPI: Protein–protein interaction network
ROC: Receiver operating characteristic
SCC: Squamous cell carcinoma
SNP: Single Nucleotide Polymorphism
SNV: Single nucleotide variant
STRING: Search Tool for the Retrieval of Interacting Genes database
TMB: Tumor mutation burden
UCEC: Uterine corpus endometrial carcinoma
